# Supplementary material for: Suppressing electrolyte-lithium metal reactivity via Li+-desolvation in uniform nano-porous separator
Source: Nat Commun. 2022 Jan 10;13:172. doi: 10.1038/s41467-021-27841-0 (PMC8748786; doi:10.1038/s41467-021-27841-0)
Supplement: Supplementary file 1 — Supplementary Information [file 41467_2021_27841_MOESM1_ESM.pdf]

Supplementary information

Suppressing Electrolyte-Lithium Metal Reactivity via Li^+-Desolvation in Uniform Nano-Porous Separator

Li Sheng[1], Qianqian Wang[1], Xiang Liu[2], Hao Cui[1], Xiaolin Wang[1], Yulong Xu[1], Zonglong Li[1], Li Wang[1], Zonghai Chen[2], Gui-Liang Xu[2], Jianlong Wang[1], Yaping Tang[1], Khalil Amine[2,3], Hong Xu[1,]* and Xiangming He[1,]*

[1] Institute of Nuclear and New Energy Technology, Tsinghua University, Beijing 100084, P. R. China.

[2] Chemical Sciences and Engineering Division, Argonne National Laboratory, Lemont, IL 60439, USA.

[3] Materials Science and Engineering, Stanford University, Stanford, CA 94305, USA.

*Corresponding authors: hongxu@tsinghua.edu.cn; hexm@tsinghua.edu.cn

Table of contents

Supplemental Experimental Procedures	Page 2-3
Supplementary Figures 1-30	Page 4-33
Supplementary Tables 1-17	Page 34-61
Supplementary References	Page 62

Supplemental Experimental Procedures

Materials

Zirconium tetrachloride ($ZrCl_4$), 1,4-benzene dicarboxylic acid, Zirconium (IV) n-propoxide ($Zr(OPr)_4$), methacrylic acid, propylene glycol methyl ether acetate (PGMEA), poly(vinylidene difluoride) (PVDF) and N-methyl-2-pyrrolidone (NMP) were obtained from TCI. Tetrahydrofuran (THF), *N*, *N*-dimethylformamide (DMF) and methanol were obtained from Xilong Chemicals. Dimethyl carbonate (DMC) (Sigma-Aldrich, 99.9%) was dried with 4 Å molecular sieves before use. Commercial Li metal foil was purchased from China Energy Lithium Co., LTD. Commercial copper foil was purchased from Kejing Co. LTD. (Hefei, China), which was treated according to the report[1] before utilization. $LiNi_{0.6}Mn_{0.2}Co_{0.2}O_2$ (NMC622) was kindly provided by Beijing Easpring Material Technology Co., LTD.

The microporous polypropylene (PP) separator (Celgard 2500) was provided by Celgard Inc.; the liquid electrolyte was manufactured by the Beijing Institute of Chemical Reagents Co., Ltd. The electrolyte used was 1.0 M $LiPF_6$ in a 1/1/1 (w/w/w) mixture of ethylene carbonate (EC), dimethyl carbonate (DMC) and ethyl methyl carbonate (EMC) and 1 wt% of vinylene carbonate (VC) as an additive. The electrolyte was stored in a glove box under dry argon; the oxygen and water contents were controlled to below 0.2 ppm.

X-ray crystallographic data for Zr-MOC (CCDC No: 2022033) has been deposited at Cambridge Crystallographic Data Centre, 12 Union Road, Cambridge CB21ES, UK; fax: (+44) 1223-336-033. Data can be obtained free of charge from the Cambridge Crystallographic Data Centre via the Internet at www.ccdc.cam.ac.uk/data_request/cif using the CCDC number given above.

Lithographic performance evaluation

The Zr-MOC with 12 methacrylic acid ligands was synthesized according to Supplementary Fig. 1. The colourless crystals were obtained with a yield of 87%, which show high solubility in organic solvents. Spin-coating of the Zr-MOC solution, which dissolved the cluster in propylene glycol methyl ether acetate (PGMEA) to obtain 5wt% solution, giving out a smooth film, and good patterns can be achieved under 365 nm UV exposure (Supplementary Figs. 2-4).

Preparation of Zr-MOC@PP-2 separator

The Zr-MOC was dissolved in PGMEA to give a 50 wt/wt% solution. After that, the PP separator was immersed in the mixture, and the soaked PP separator was taken out, dried in the glovebox. Before assembling a coin cell, the dried separator was immersed in the $LiPF_6$-LE for 24 h, according to the Zr-MOCN@PP.

Preparation of Zr-MOC@PP-3 separator

The Zr-MOC was dissolved in PGMEA to give a 50 wt/wt% solution. After that, the PP separator was immersed in the mixture, and the soaked PP separator was taken out, dried in the glovebox. The prepared separators were directly added $LiPF_6$-LE electrolyte and assembled to a coin cell.

Electrochemical Measurements

The Li plating/stripping Coulombic efficiency (CE) was evaluated by using Li | Cu asymmetric cells with the similar protocol in the previous report[2]. Specially, the Li plating Q_1 (2 mAh cm^{-2}) was used for Li plating/stripping (Q_2, 1 mAh cm^{-2}) for n cycles. A final Li stripping Q_3 exhausted all Li in the Cu current collector. The CE value was calculated as the following Equation S1:

$$CE = \frac{n\,Q_2 + Q_3}{n\,Q_2 + Q_1} \times 100\% \qquad (1).$$

Li^+ transference number (t_{Li+}) measurement was carried out by using Li symmetric cell. A constant dc bias was set as 10 mV. Therefore, t_{Li+} can be calculated from Equation S2:

$$t_{Li+} = \frac{i_1(\Delta V - i_0 R_0')}{i_0(\Delta V - i_1 R_1')} \qquad (2)$$

In Equation S2, ΔV is the polarization voltage, and i is the current; the subscripts 0 and 1 indicate initial values and steady-state values, respectively, and R' is the sum of the charge transfer resistance and the passivating film resistance; R_0' and R_1' can be obtained from two impedance spectra on the cell in the frequency range between 0.1 Hz and 10 Hz before the polarization, and after the steady state has been reached.

Impedance analyses of the Li-symmetric cells with PP separator and Zr-MOCN@PP were performed on a CHI660E Electrochemical Workstation (Shanghai Chenhua) with electrochemical impedance spectroscopy (EIS). The perturbation amplitude was 10 mV and the frequency range from 0.1 Hz to 10 kHz at room temperature.

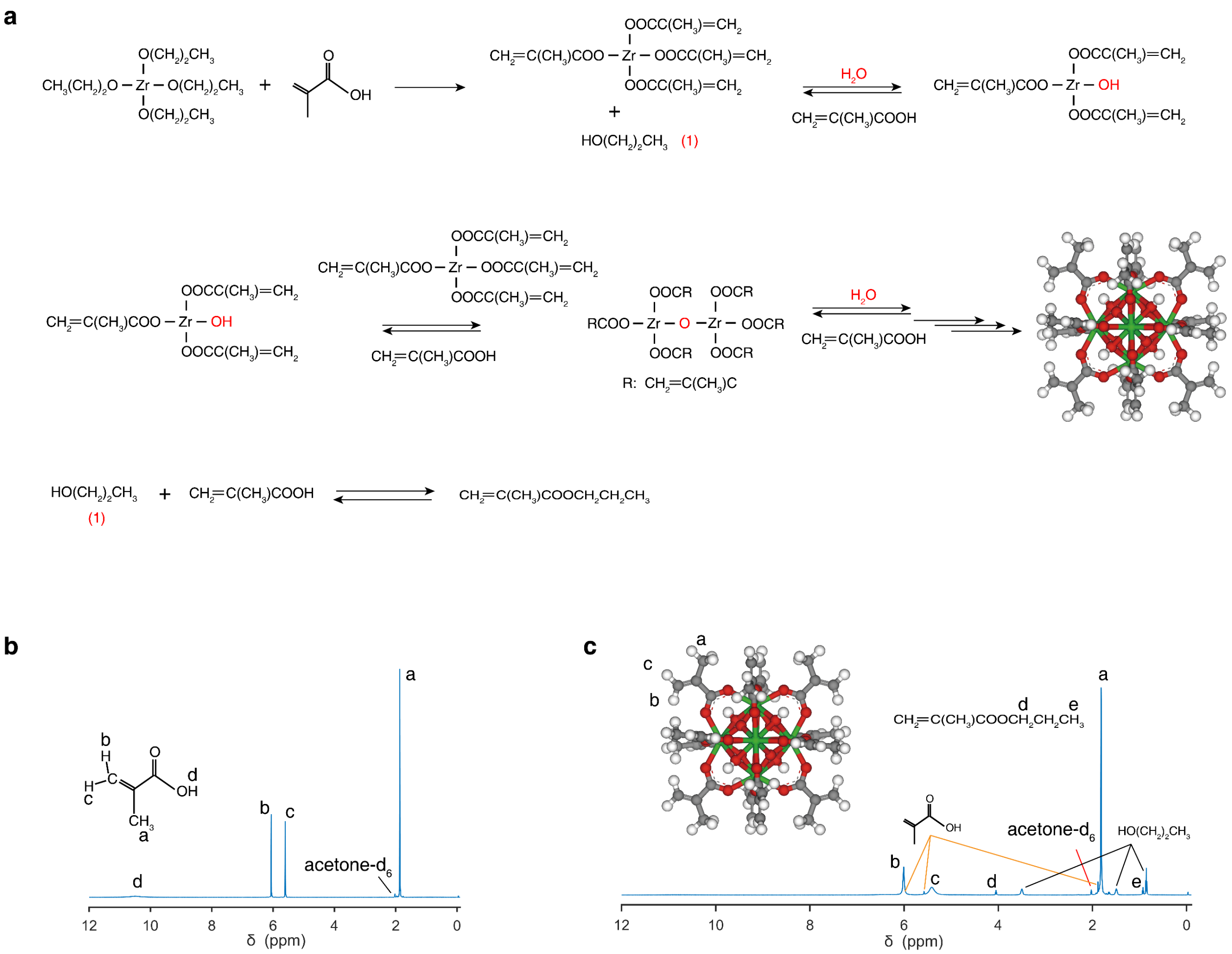

Supplementary Figure 1 | Preparation and characterization of metal-organic cluster (Zr-MOC). a, Preparation scheme of Zr-MOC. **b**, **c**, ^{1}H NMR spectra of methacrylic acid and Zr-MOC, respectively.

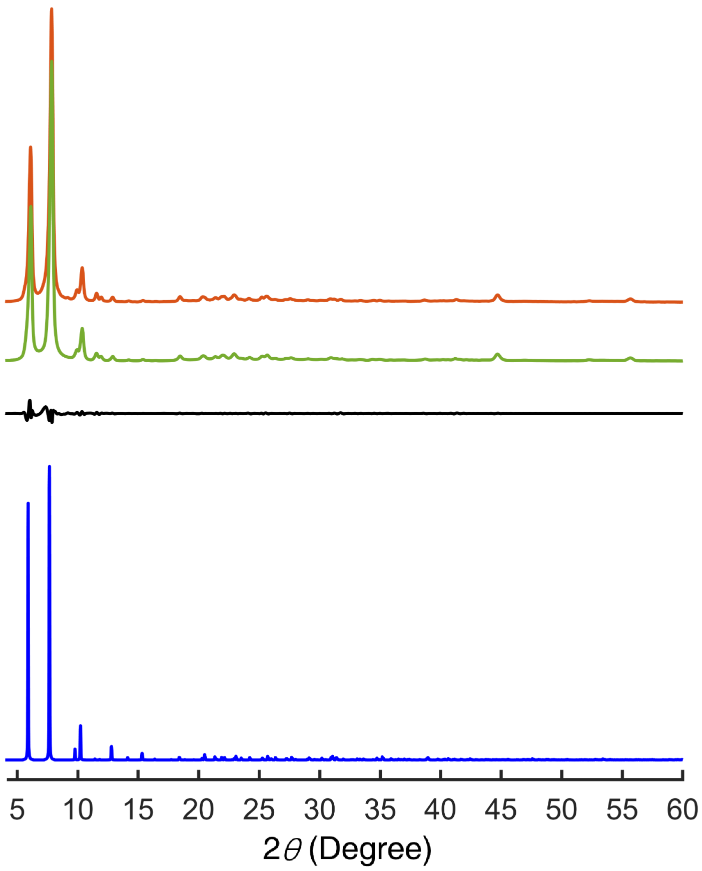

Supplementary Figure 2 | PXRD profiles of Zr-MOC (red: experimentally observed; green: Rieveld refined; black: their difference; blue: theoretical data) with R_{wp} = 6.71% and R_p = 4.60%.

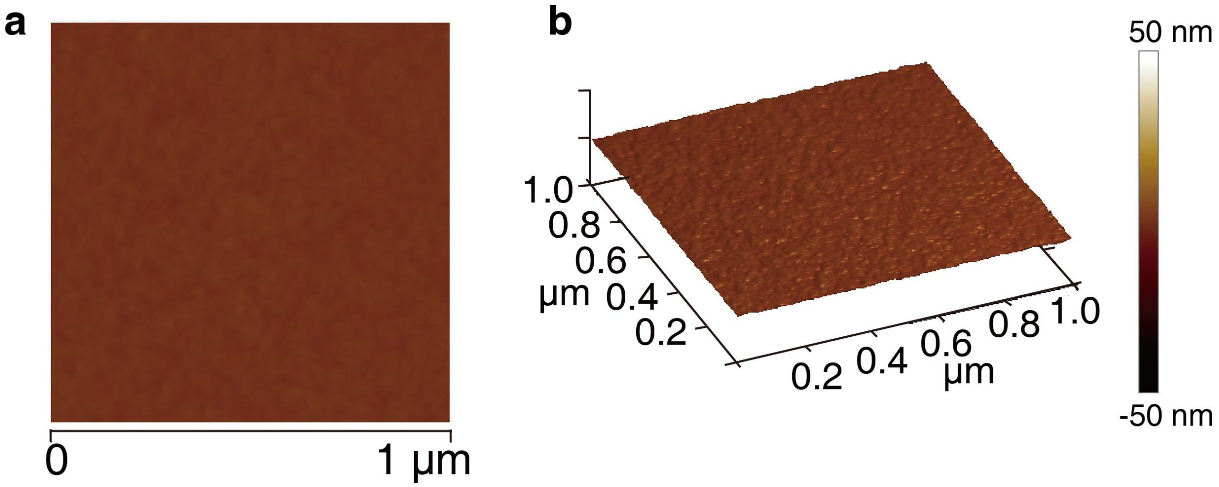

Supplementary Figure 3 | AFM images (1 X 1 μm) of metal-organic cluster (Zr-MOC) film prepared by spin-coating. a, Top view. **b**, Cross-section view.

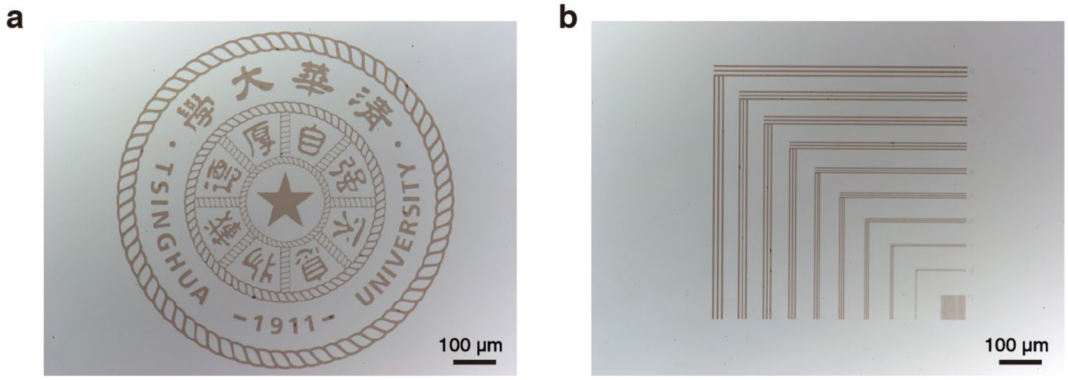

Supplementary Figure 4 | Optical microscope images of patterned Zr-MOC-based photoresist. a, Tsinghua university-logo pattern. **b**, Line-and-space pattern.

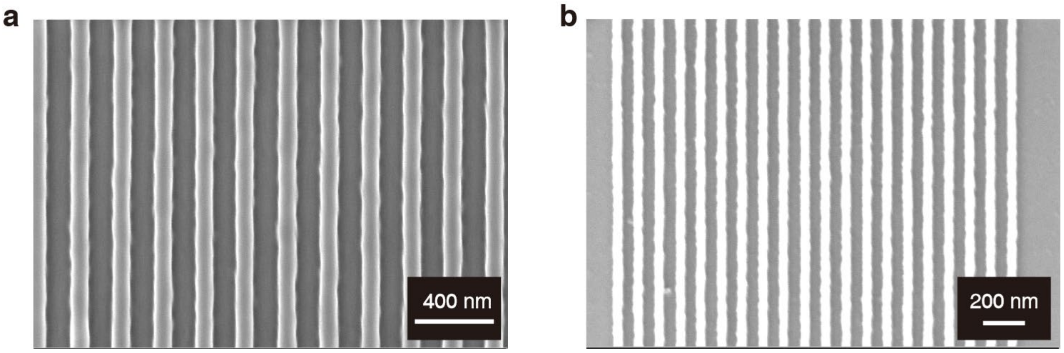

Supplementary Figure 5 | Scanning electron microscope images of patterned Zr-MOC-based photoresist.

a, Scale bar: 400 nm. **b**, scale bar: 200 nm.

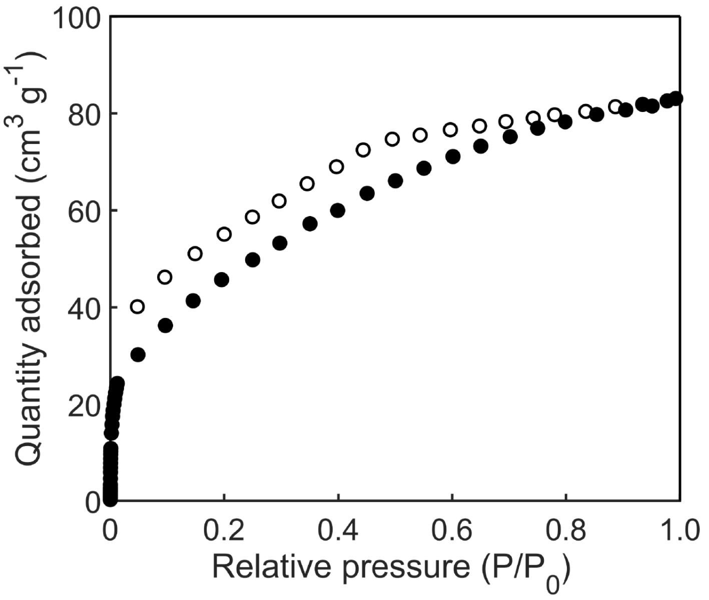

Supplementary Figure 6 | Nitrogen-sorption isotherm curves of Zr-MOCN measured at 77 K.

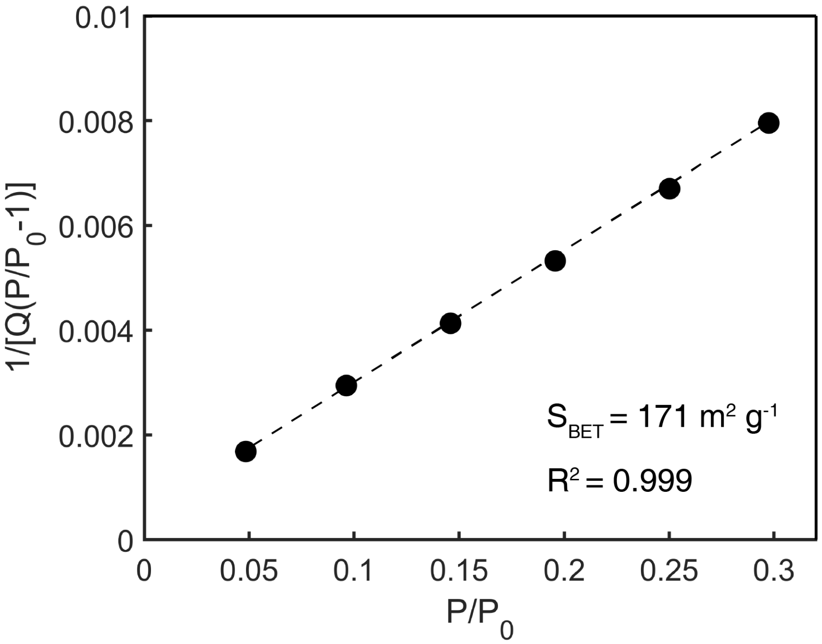

Supplementary Figure 7 | BET plot of Zr-MOCN calculated from N_2 adsorption isotherm at 77 K.

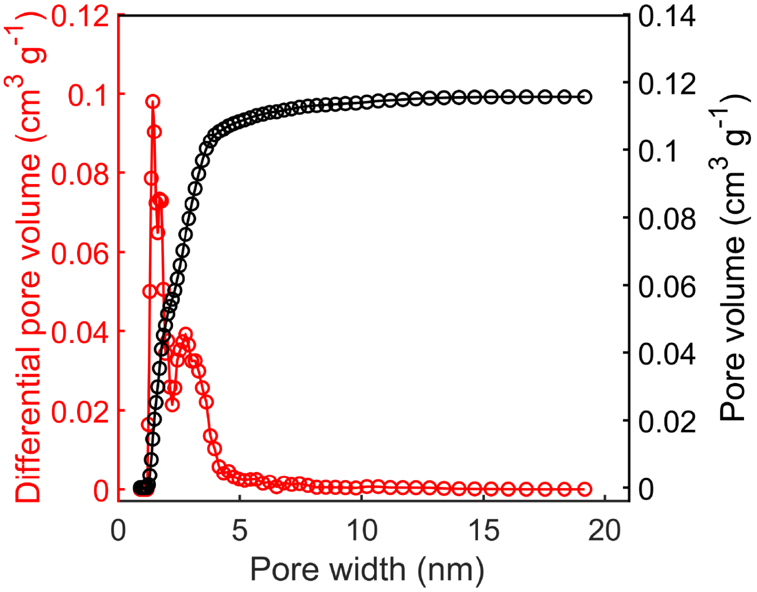

Supplementary Figure 8 | Pore size distribution (red) and cumulative pore volume (black) profiles of Zr-MOCN.

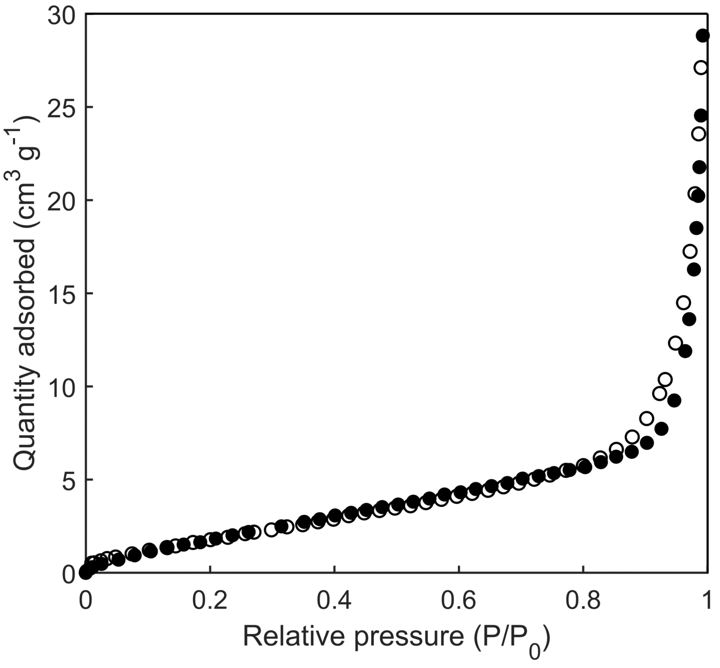

Supplementary Figure 9 | Nitrogen-sorption isotherm curves of Zr-MOC measured at 77 K.

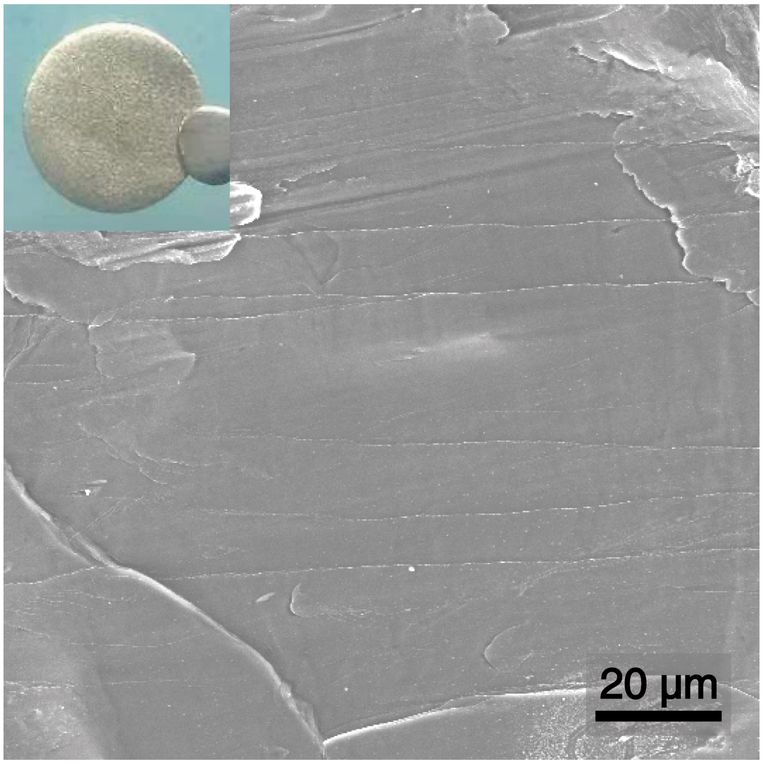

Supplementary Figure 10 | Top-view SEM images of the pristine lithium metal. The inset shows the photo of the pristine lithium metal.

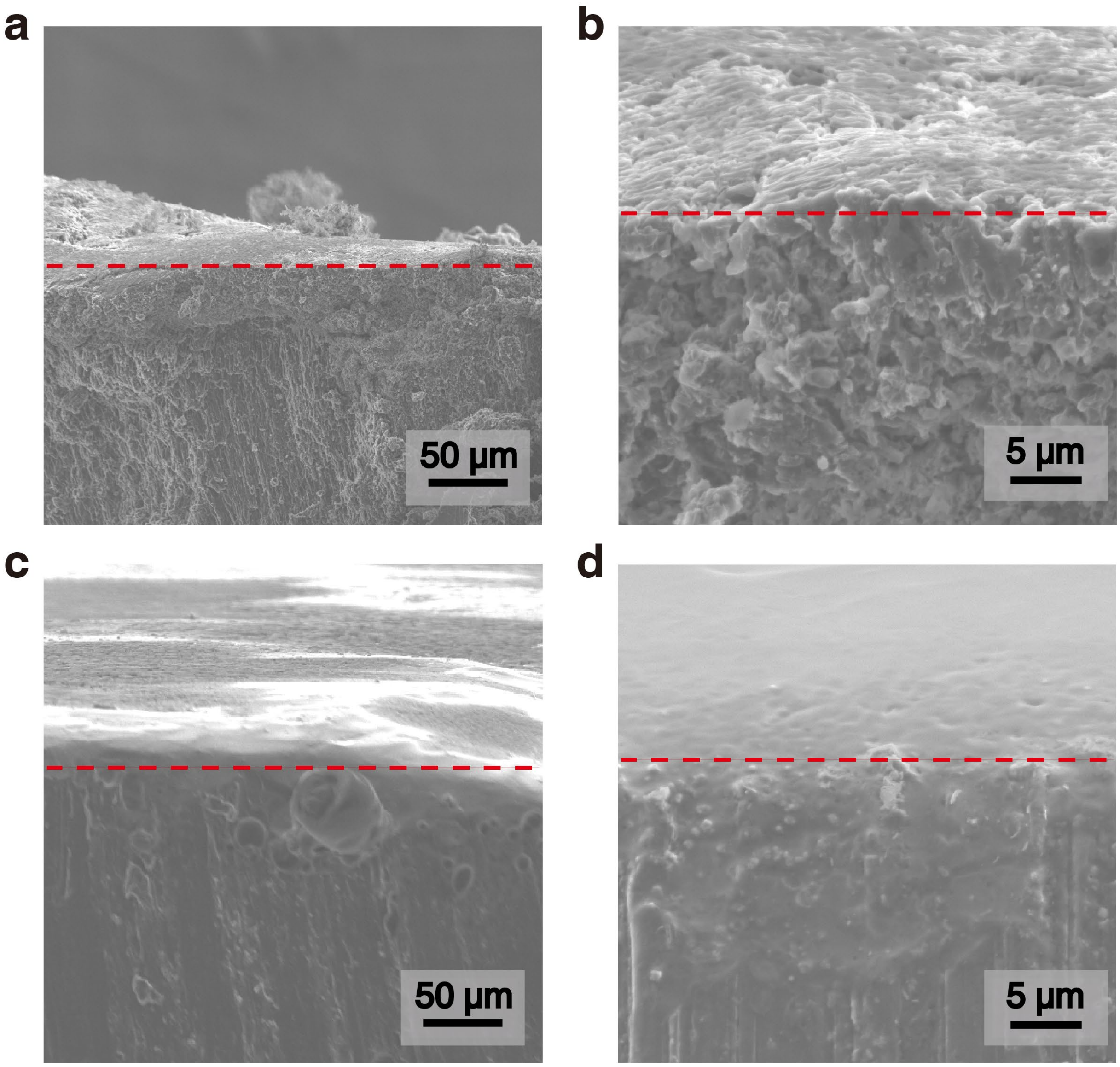

Supplementary Figure 11 | Cross-sectional SEM images of the lithium metal electrode at the current density of 1.0 mA cm^{-2} with an areal capacity of 1.0 mAh cm^{-2} after 340 h. **a**, **b**, the PP separator-based symmetric lithium cell. **c**, **d**, Zr-MOCN@PP-based symmetric lithium cell.

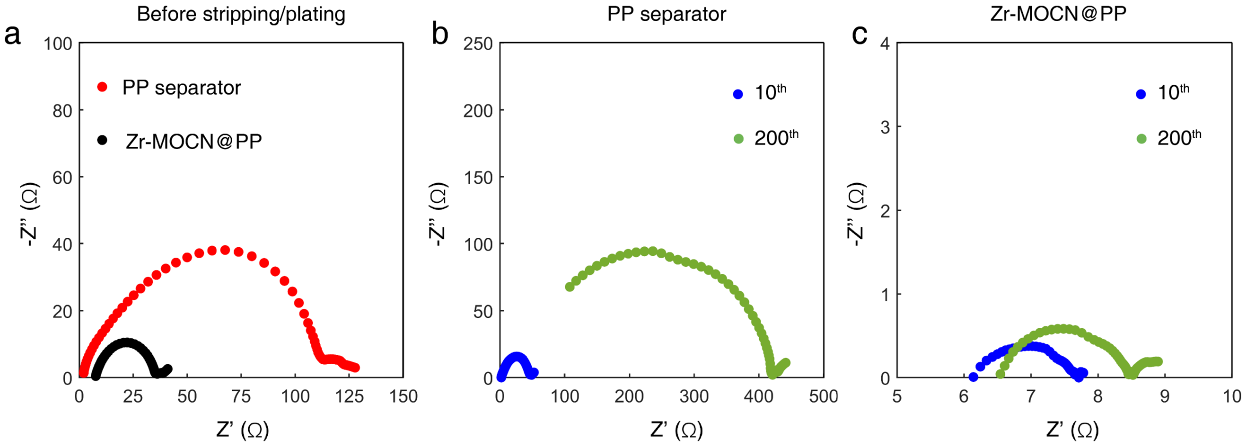

Supplementary Figure 12 | Impedance spectroscopy of Li-symmetric cells with the PP separator and Zr-MOCN@PP before and after stripping/plating at a current density of 1 mA cm^{-2}, respectively. a, Before stripping/plating. **b**, **c**, after 10th, 200th stripping/plating of the PP separator and Zr-MOCN@PP based cells, respectively.

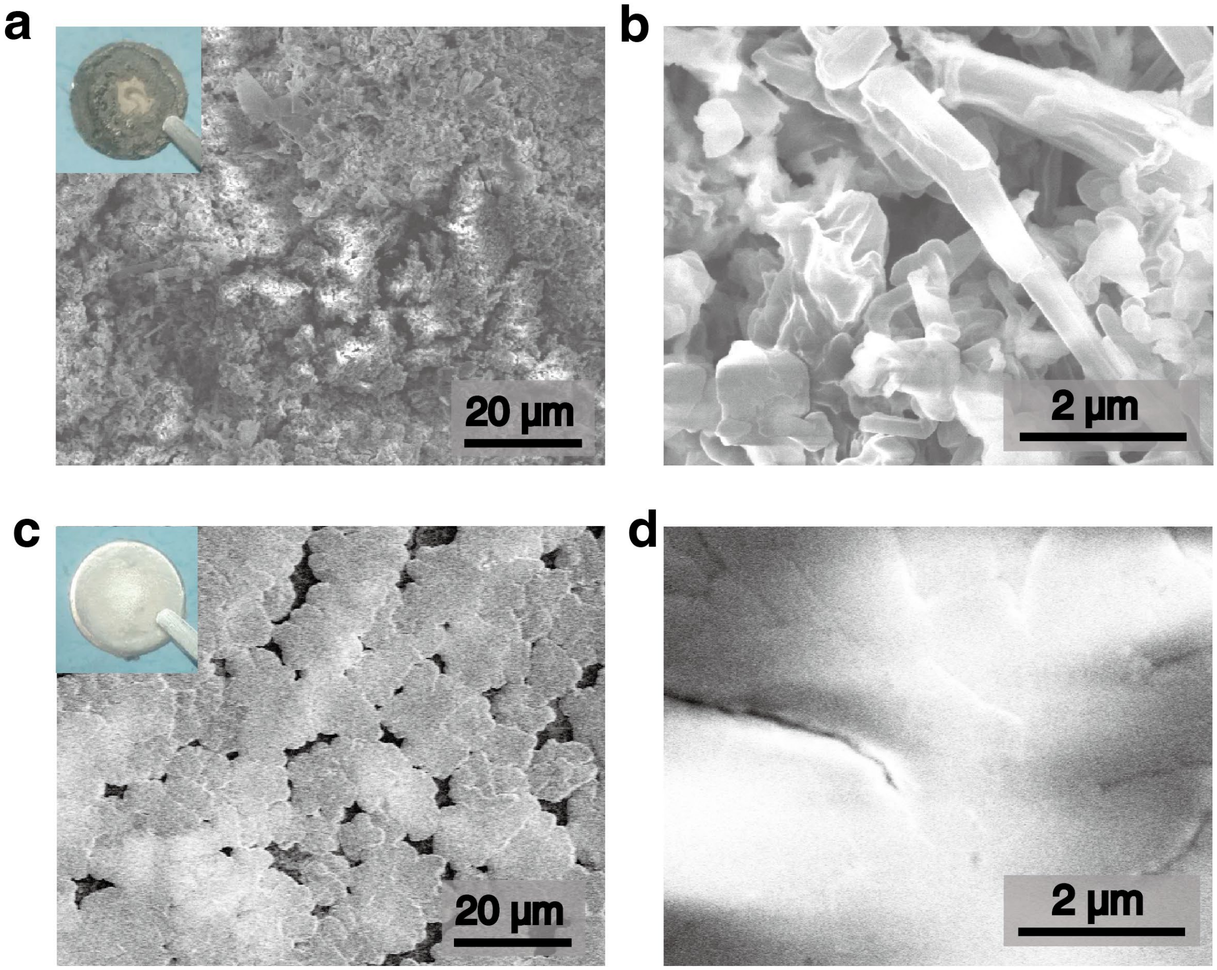

Supplementary Figure 13 | Top-view SEM images of the lithium metal electrode at the current density of 10 mA cm^{-2} with an areal capacity of 10 mAh cm^{-2} after 100 h cycling. a, **b**, the PP separator-based symmetric lithium cell. **c**, **d**, Zr-MOCN@PP-based symmetric lithium cell.

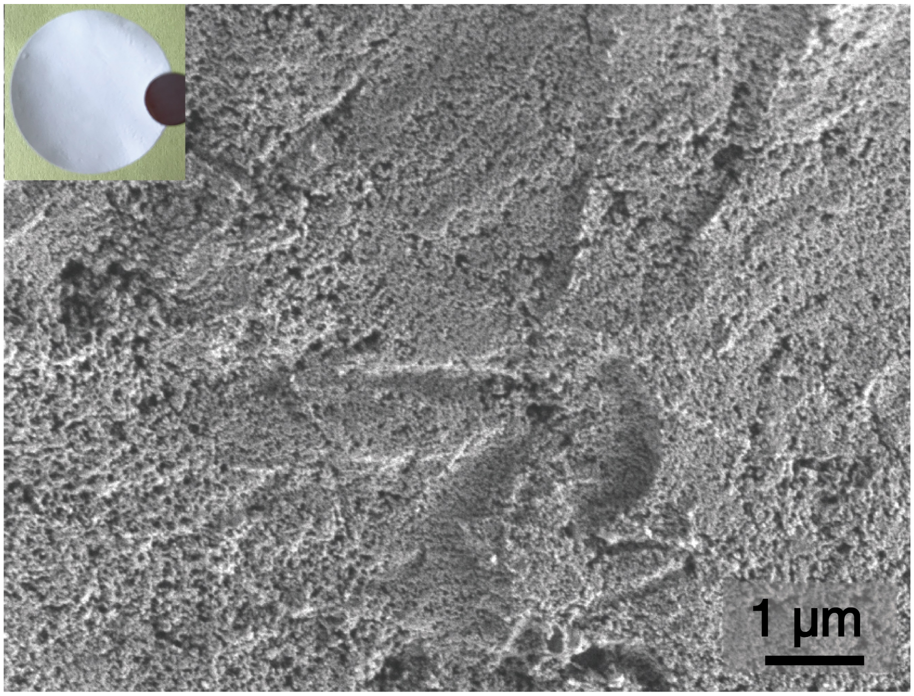

Supplementary Figure 14 | SEM images of Zr-MOC@PP. The inset is the photo of the Zr-MOC@PP membrane.

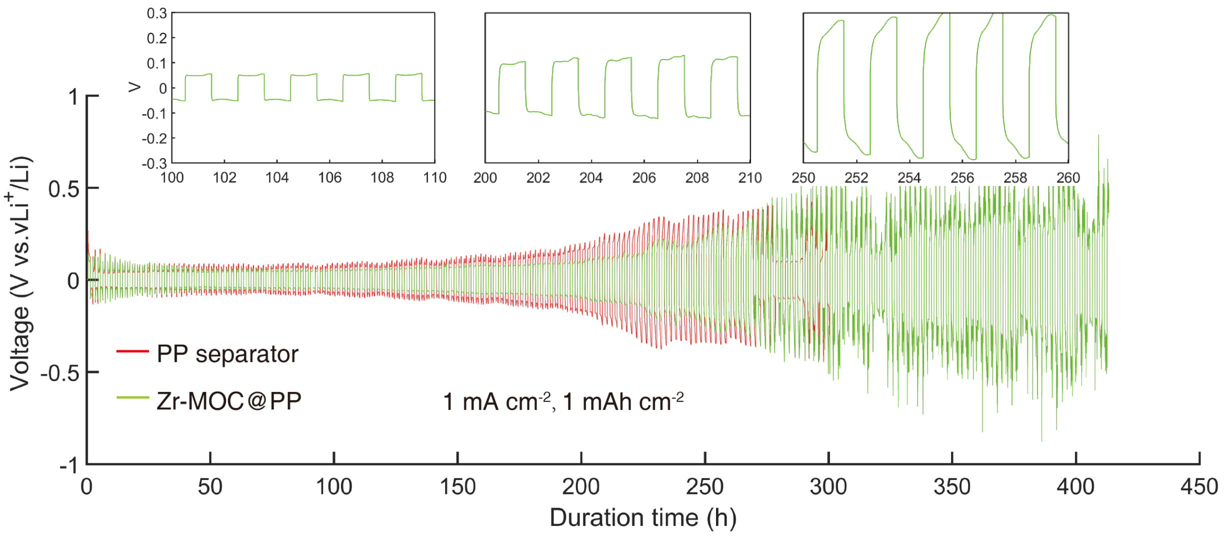

Supplementary Figure 15 | Voltage versus time for symmetric lithium cell where each half-cycle lasts 1 h. Initial voltage profiles of the PP separator (red curve) and Zr-MOC@PP (green curve)-based cell at a fixed current density of 1.0 mA cm^{-2} (1.0 mAh cm^{-2}).

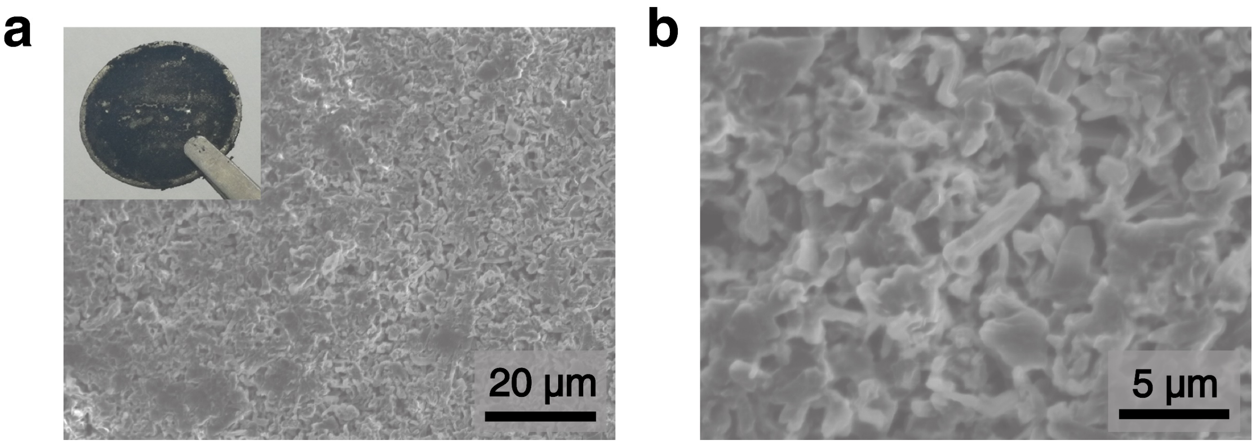

Supplementary Figure 16 | SEM images of the lithium metal anode after 120 h cycling at 1.0 mA cm^{-2}, 1.0 mAh cm^{-2} in Zr-MOC@PP-based symmetric lithium cell. a, Scale bar: 20 µm. **b**, scale bar: 5 µm. The insets show the lithium metal after 120 h cycling.

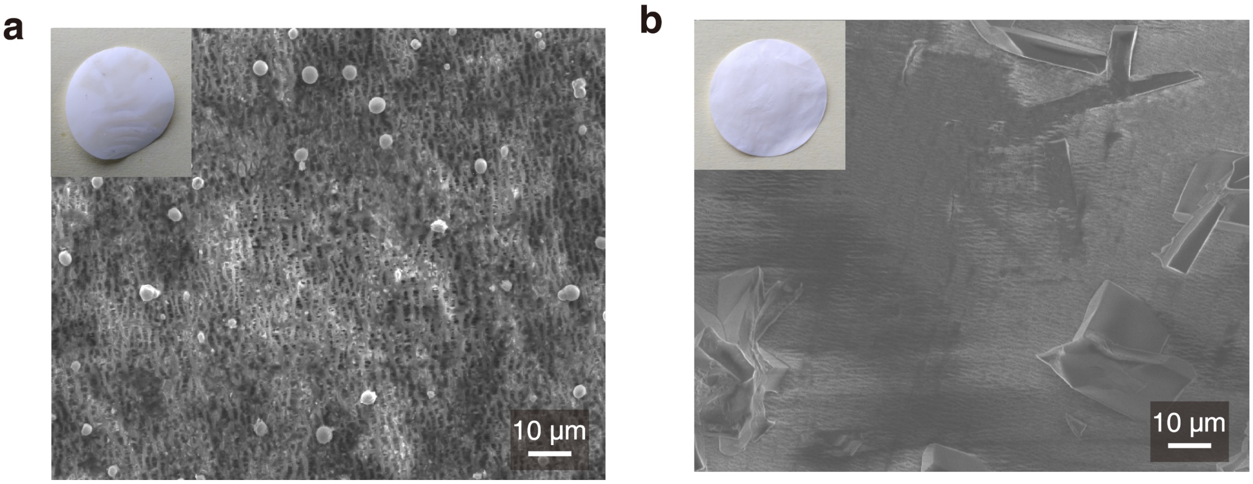

Supplementary Figure 17 | SEM images of **a**, Zr-MOC@PP-2; **b**, Zr-MOC@PP-3. The inset is the photo of the Zr-MOC@PP-2 and Zr-MOC@PP-3 membranes, respectively.

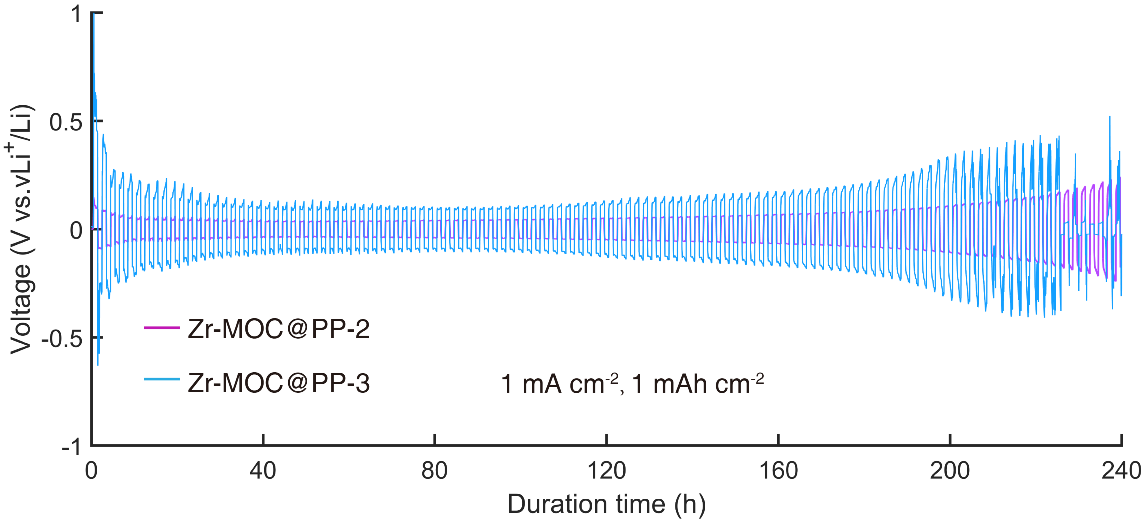

Supplementary Figure 18 | Voltage versus time for symmetric lithium cell where each half-cycle lasts 1 h. Initial voltage profiles of the Zr-MOC@PP-2 separator (purple curve) and Zr-MOC@PP-3 (light blue curve)-based cell at a fixed current density of 1.0 mA cm^{-2} (1.0 mAh cm^{-2}).

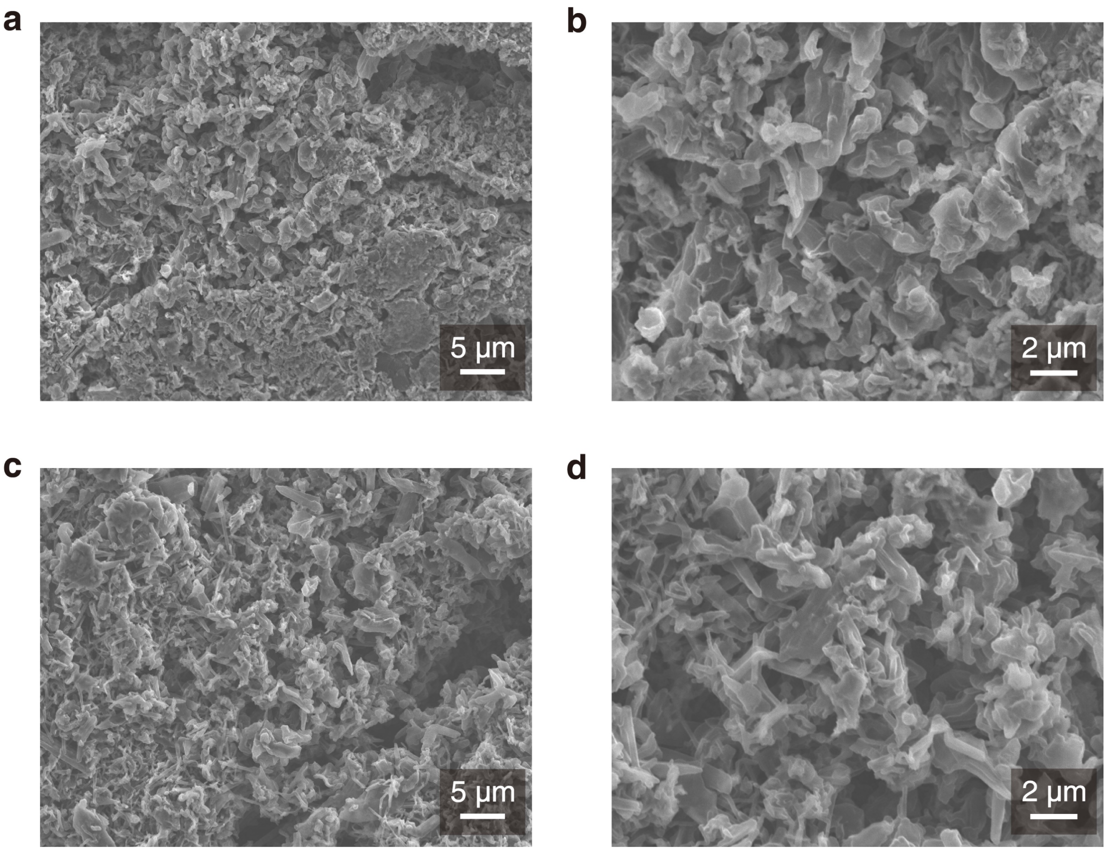

Supplementary Figure 19 | SEM images of the lithium metal anode after 120 h cycling at 1.0 mA cm^{-2}, 1.0 mAh cm^{-2} in the different separators based symmetric lithium cells. **a**, **b**, Zr-MOC@PP-2. **c**, **d**, Zr-MOC@PP-3.

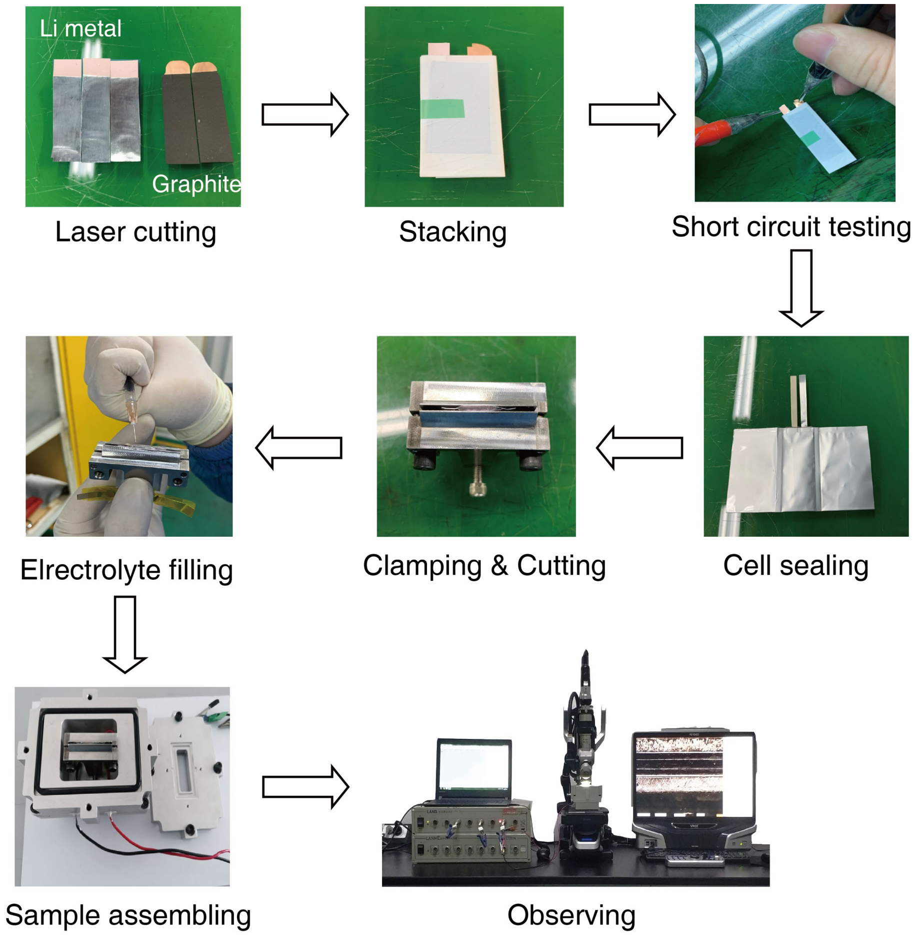

Supplementary Figure 20 | Scheme of the sample preparation for In-situ optical microscopy observation.

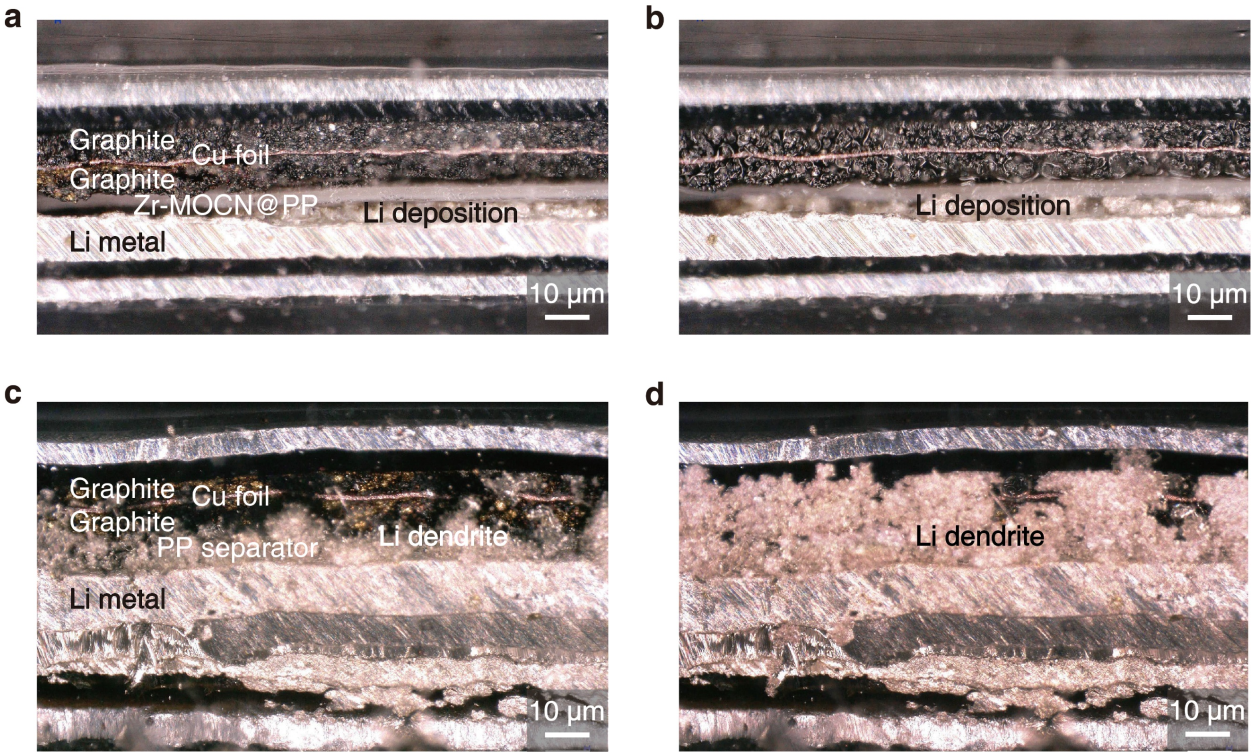

Supplementary Figure 21 | Observation of Li-deposition morphology by in-situ optical microscopy. a, **b**, The Zr-MOCN@PP based cell discharged at a current density of 1.0 mA cm^{-2} after 500 s and 2000 s, respectively. **c**, **d**, The PP separator based cell discharged at a current density of 1.0 mA cm^{-2} after 500 s and 2000 s, respectively.

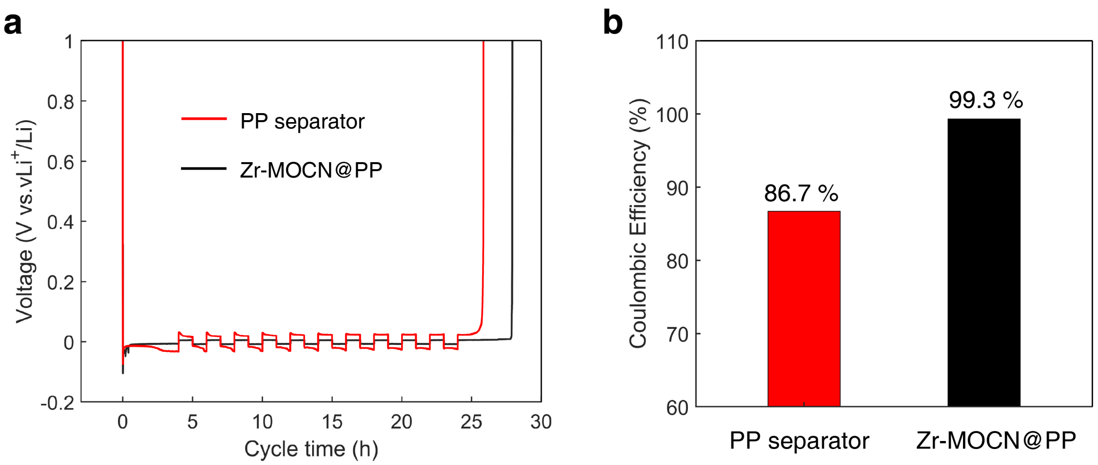

Supplementary Figure 22 | Coulombic efficiency of Li plating/stripping. a, Li plating/stripping profiles and **b**, Coulombic efficiency in the PP separator and Zr-MOCN@PP based cells at a current density of 0.5 mA cm^{-2} and 0.5 mAh cm^{-2}.

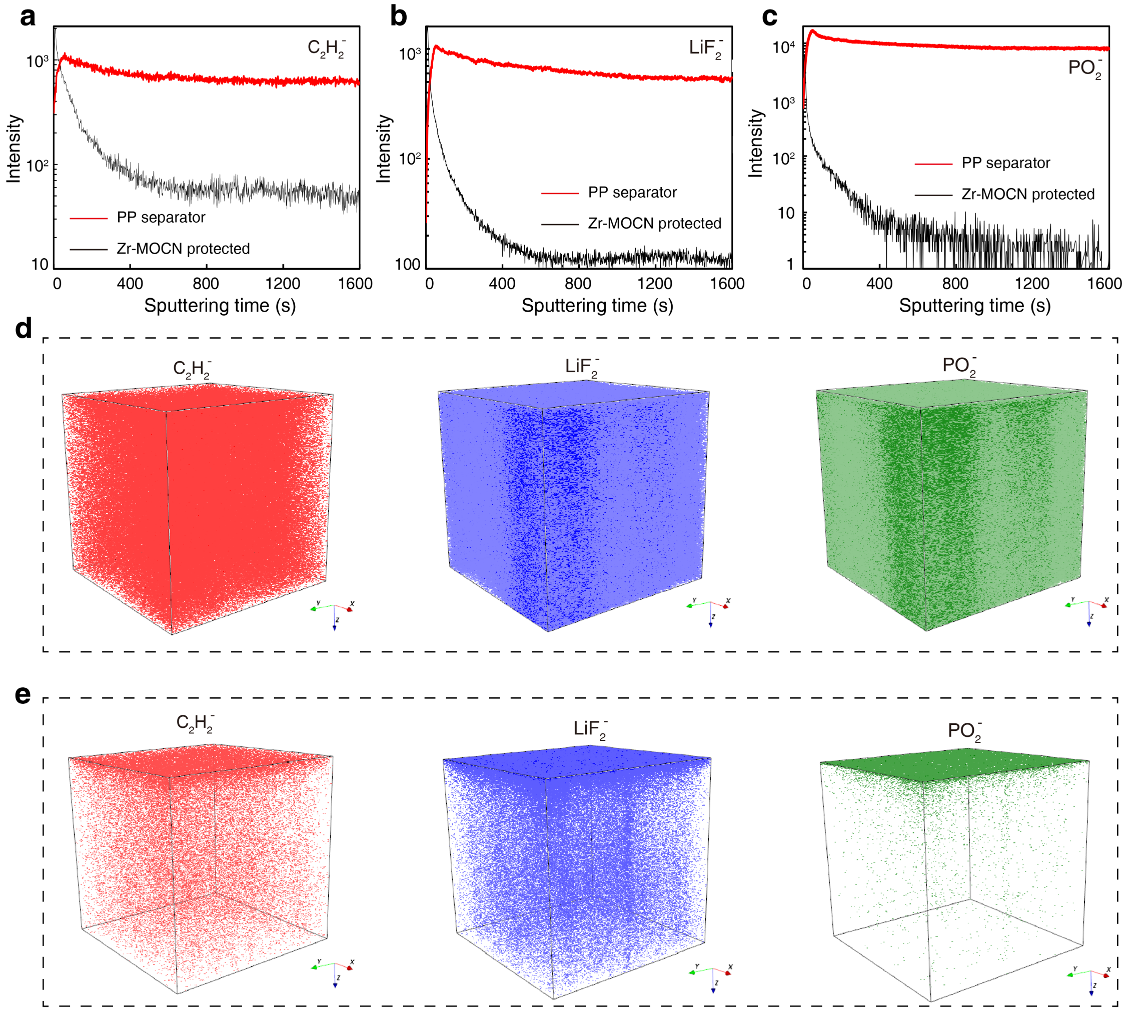

Supplementary Figure 23 | Characterization of chemical compositions on the cycled lithium metal electrodes by ToF-SIMS. a, **b**, **c** ToF-SIMS depth profiling of several typical second ion fragments on the cycled lithium metal electrode surface of the pristine PP and Zr-MOCN protected areas, respectively. **d**, **e** ToF-SIMS 3D render of second ion fragments with 1600 s of sputtering in the lithium metal electrodes cycled in the PP and Zr-MOCN protected areas, respectively.

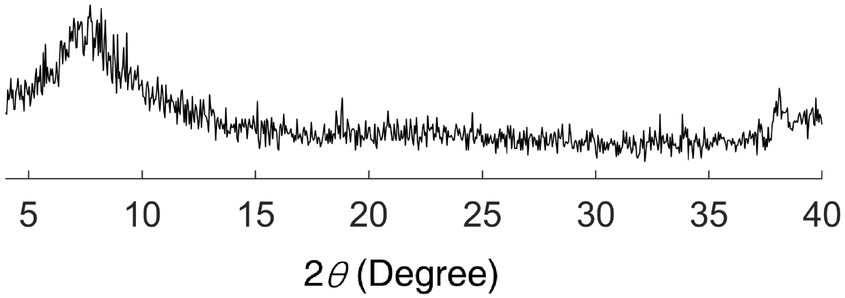

**Supplementary Figure 24 | ** PXRD profiles of Zr-MOCN.

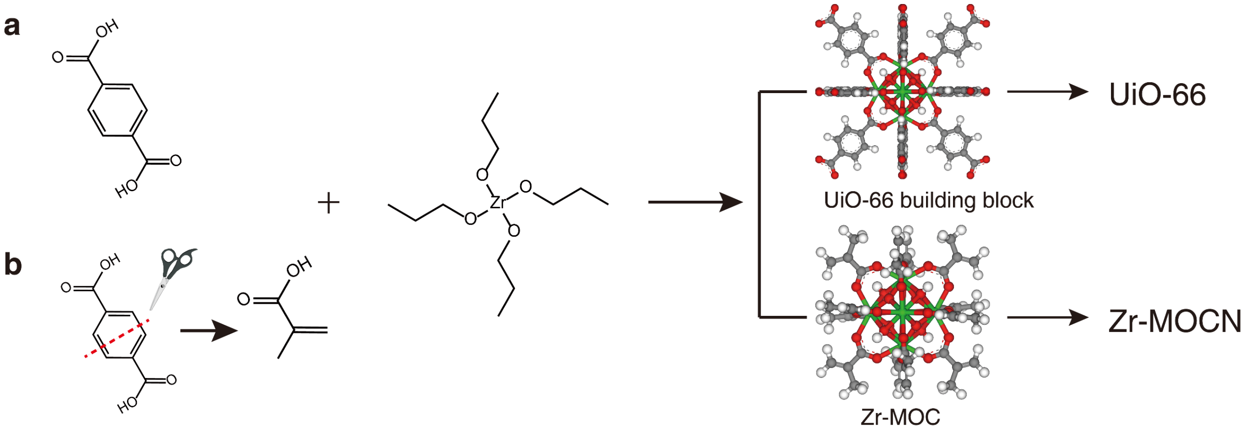

Supplementary Figure 25 │ The structure of UiO-66 and Zr-MOCN. a, **b**, Synthesis scheme of UiO-66 and Zr-MOCN, respectively.

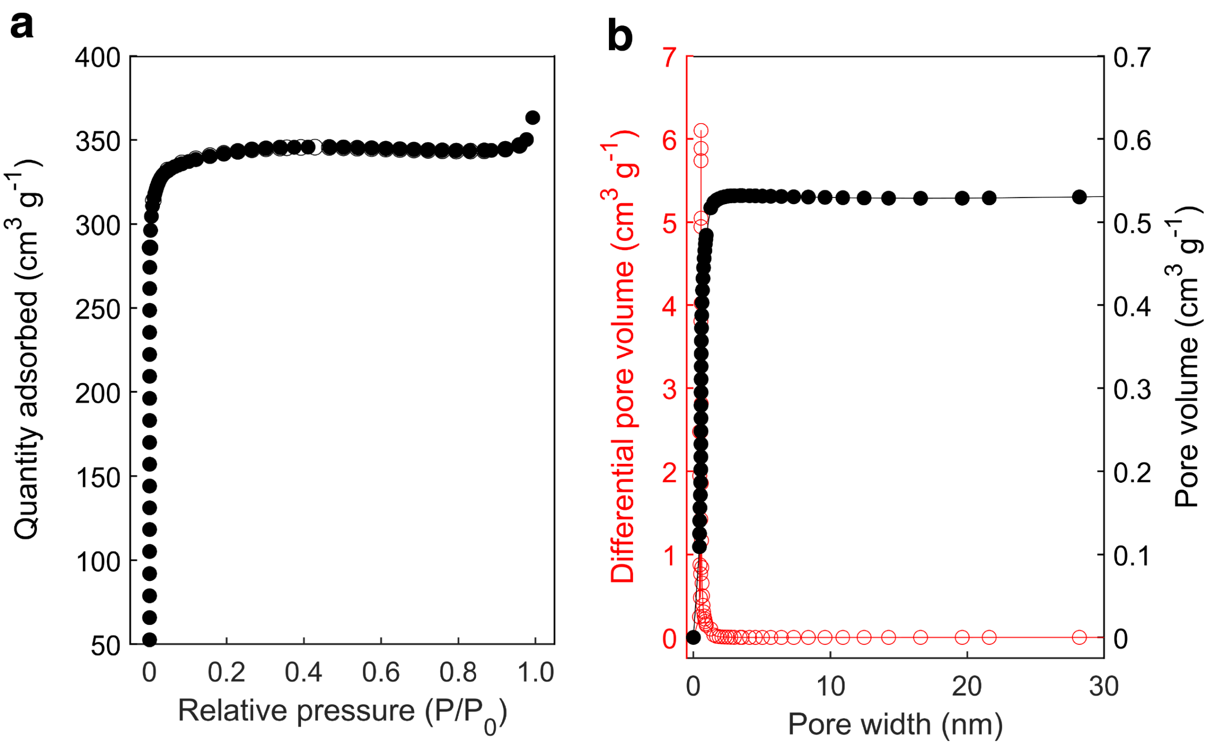

Supplementary Figure 26 | Porosity characterization of UiO-66. a, Nitrogen-sorption isotherm curves measured at 77 K. **b**, Pore size distribution (red) and cumulative pore volume (black) profiles.

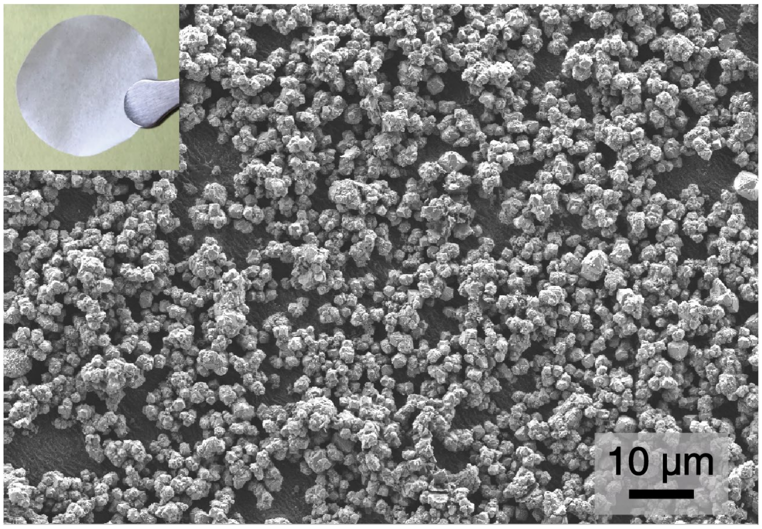

Supplementary Figure 27 | SEM images of UiO-66@PP. The inset is the photo of the UiO-66@PP membrane.

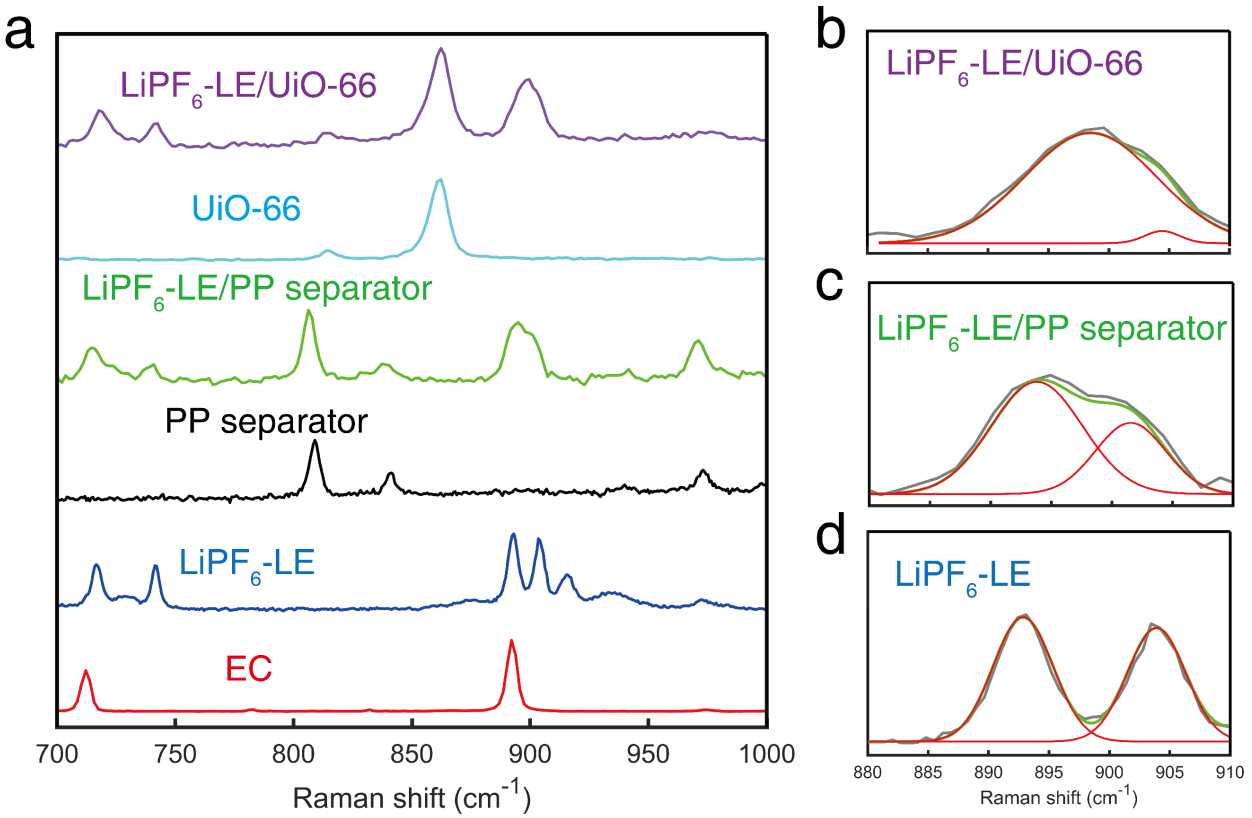

Supplementary Figure 28 | Raman spectra at room temperature. a, Raman spectra of EC, $LiPF_6$-LE, PP separator, $LiPF_6$-LE/PP separator (the PP separator absorbed $LiPF_6$-LE), UiO-66, $LiPF_6$-LE/UiO-66 (UiO-66 absorbed $LiPF_6$-LE). **b**, **c**, **d**, The enlarged portion of 880 cm^{-1} to 910 cm^{-1} in (A) of $LiPF_6$-LE/UiO-66, $LiPF_6$-LE/PP separator and $LiPF_6$-LE, respectively.

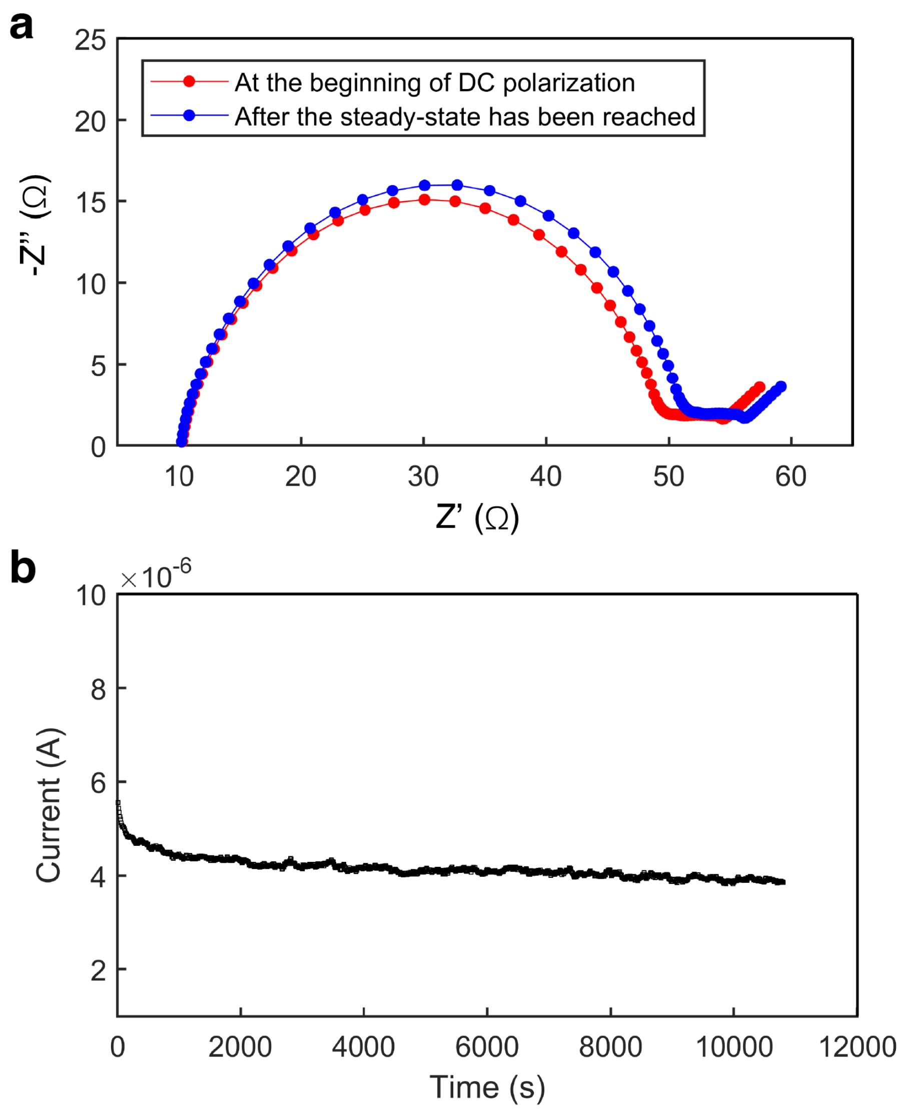

Supplementary Figure 29 | The transference number measurement. a, Impedance spectra of the Zr-MOCN@PP based cell with $LiPF_6$-LE before polarization and after the steady-state has been reached. **b**, Polarization curve of the same cell.

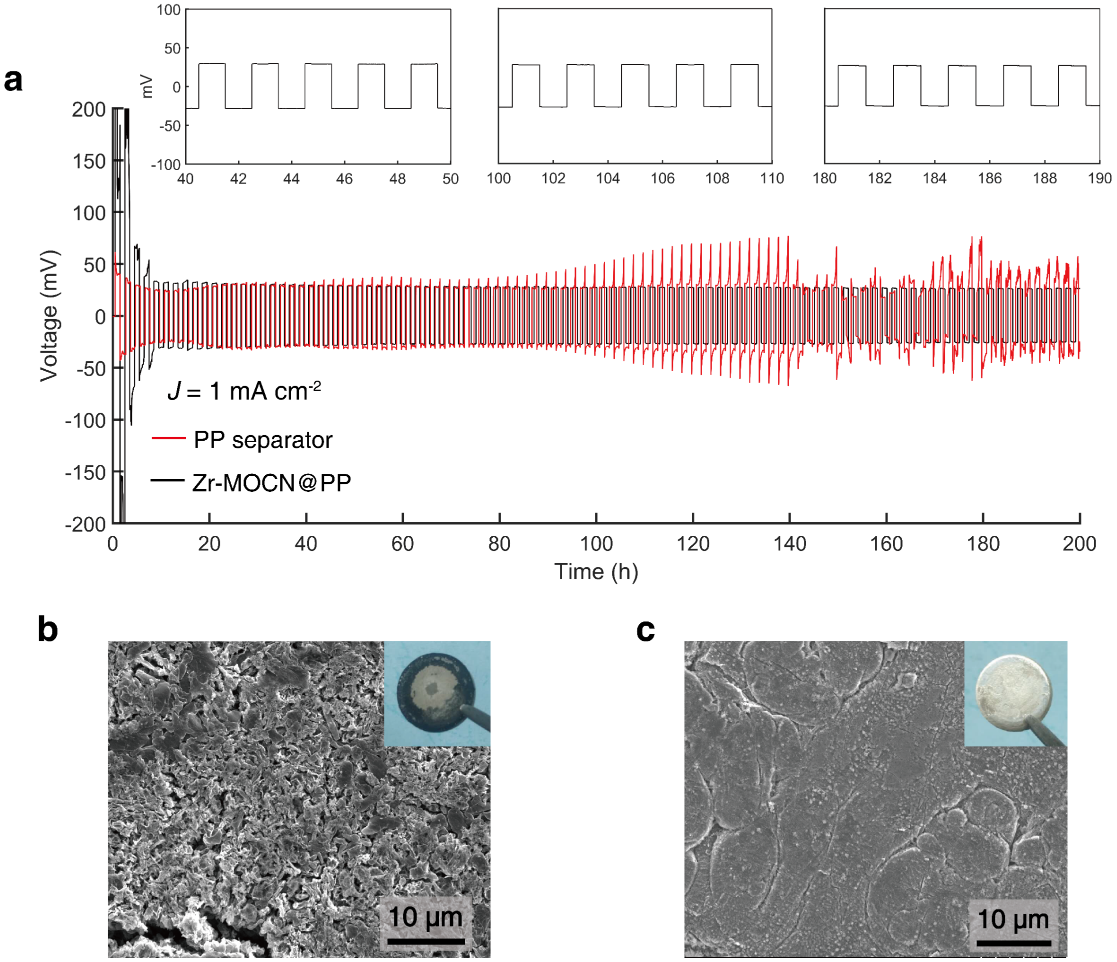

Supplementary Figure 30 | Voltage versus time for symmetric lithium cell with the ester-based electrolyte and SEM images of the lithium metal anode after 200 h cycling at 1.0 mA cm^{-2}, 1.0 mAh cm^{-2}. a, The each half-cycle lasts 1 h. Initial voltage profiles of the PP separator (red curve), Zr-MOCN@PP (red curve)-based cells at a fixed current density of 1 mA cm^{-2} (1 mAh cm^{-2}), the liquid electrolyte was 0.5 M $LiCF_3SO_3$-0.5 M $LiNO_3$ in the 1,3-dioxolane (DOL) and 1,2-dimethoxyethane (DME) (1/1, v/v). **b**, the PP separator-based symmetric lithium cell. **c**, Zr-MOCN@PP-based symmetric lithium cell. The insets show the lithium metal after 200 h cycling in different cells: the PP separator and Zr-MOCN@PP-based symmetric lithium cells, respectively.

Supplementary Table 1 | Crystal data and structure refinement for Zr-MOC (CCDC No: 2022033)

Identification code	Zr-MOC (CCDC No: 2022033)	
Empirical formula	C48 H84 O32 Zr6	
Formula weight	1720.43	
Temperature	173.00(10) K	
Wavelength	0.71073 Å	
Crystal system	Hexagonal	
Space group	$P6_3mc$	
Unit cell dimensions	a = 17.3180(8) Å	a= 90°.
	b = 17.3180(8) Å	b= 90°.
	c = 18.1144(6) Å	g = 120°.
Volume	4704.9(5) $Å^3$	
Z	2.00004	
Density (calculated)	1.214 Mg/m^3	
Absorption coefficient	0.700 mm^{-1}	
F(000)	1736	
Crystal size	0.45 x 0.35 x 0.2 mm^3	
Theta range for data collection	3.255 to 29.633°.	
Index ranges	-23<=h<=23, -19<=k<=22, -25<=l<=24	
Reflections collected	45260	
Independent reflections	4590 [R(int) = 0.0706]	
Completeness to theta = 25.242°	99.6 %	
Absorption correction	Semi-empirical from equivalents	
Max. and min. transmission	1.00000 and 0.71762	
Refinement method	Full-matrix least-squares on F^2	
Data / restraints / parameters	4590 / 124 / 213	
Goodness-of-fit on F^2	1.026	
Final R indices [I>2sigma(I)]	R1 = 0.0599, wR2 = 0.1428	
R indices (all data)	R1 = 0.0985, wR2 = 0.1620	
Absolute structure parameter	-0.09(3)	
Extinction coefficient	n/a	
Largest diff. peak and hole	0.517 and -0.447 $e.Å^{-3}$	

**Supplementary Table 2 | ** Atomic coordinates ($\times 10^4$) and equivalent isotropic displacement parameters ($Å^2 \times 10^3$) for Zr-MOC (CCDC No: 2022033). U(eq) is defined as one third of the trace of the orthogonalized U^{ij} tensor.

	x	y	z	U(eq)
Zr(01)	7346(1)	2654(1)	4918(1)	63(1)
Zr(02)	5993(1)	1985(1)	3360(1)	68(1)
O(003)	6667	3333	3063(6)	54(3)
O(004)	6098(3)	2197(5)	4490(4)	61(2)
O(005)	8185(7)	3336(11)	5844(5)	167(5)
O(006)	5944(14)	773(18)	3681(11)	82(5)
O(007)	7420(3)	2580(3)	3615(5)	81(3)
O(008)	6667	3333	5482(5)	59(3)
O(009)	6858(16)	1185(18)	4671(14)	101(7)
O(00A)	6476(7)	1699(6)	2279(4)	110(3)
C(00C)	6382(18)	670(16)	4073(14)	89(5)
C(00E)	5715(12)	1430(20)	1113(14)	177(7)
C(00H)	5807(9)	1614(19)	1939(10)	140(5)
C(00I)	6833(18)	-397(18)	4729(18)	125(9)
C(00K)	6333(18)	1440(20)	700(10)	237(11)
C(00N)	8440(20)	4222(11)	6085(15)	203(9)
C(5)	6277(19)	-277(15)	4172(16)	106(6)
C(6)	9160(20)	4580(10)	6563(15)	219(9)
C(12)	9510(40)	4150(30)	7050(20)	232(12)
O(14)	7208(14)	1392(16)	4596(12)	80(5)
O(17)	6336(13)	907(16)	3616(11)	80(5)
C(22)	6846(16)	846(15)	4168(14)	80(5)
C(37)	6840(20)	34(15)	4234(17)	109(6)
C(45)	6130(20)	-756(17)	3823(19)	128(10)
C(20)	7460(20)	-50(20)	4724(19)	137(9)
C(46)	5540(20)	-1040(20)	3787(18)	138(11)
C(31)	9370(40)	3850(30)	6770(20)	230(12)

Supplementary Table 3 | Bond lengths [Å] and angles [°] for Zr-MOC (CCDC No: 2022033).

Zr(01)-Zr(02)	3.4762(11)
Zr(01)-Zr(02)#1	3.4762(11)
Zr(01)-O(004)#1	2.047(4)
Zr(01)-O(004)	2.047(4)
Zr(01)-O(005)	2.145(9)
Zr(01)-O(005)#2	2.145(9)
Zr(01)-O(007)	2.370(10)
Zr(01)-O(008)	2.281(4)
Zr(01)-O(009)#2	2.29(3)
Zr(01)-O(009)	2.29(3)
Zr(01)-O(14)	2.16(3)
Zr(01)-O(14)#2	2.16(3)
Zr(02)-Zr(01)#3	3.4762(11)
Zr(02)-O(003)	2.092(3)
Zr(02)-O(004)	2.070(7)
Zr(02)-O(006)#4	2.14(3)
Zr(02)-O(006)	2.14(3)
Zr(02)-O(007)	2.199(6)
Zr(02)-O(007)#3	2.199(5)
Zr(02)-O(00A)#4	2.279(7)
Zr(02)-O(00A)	2.279(7)
Zr(02)-C(00H)	2.633(19)
Zr(02)-O(17)#4	2.27(2)
Zr(02)-O(17)	2.27(2)
O(003)-Zr(02)#3	2.092(3)
O(003)-Zr(02)#1	2.092(3)
O(004)-Zr(01)#3	2.047(4)
O(005)-C(00N)	1.435(16)
O(006)-C(00C)	1.12(2)
O(007)-Zr(02)#1	2.199(5)
O(008)-Zr(01)#1	2.281(4)
O(008)-Zr(01)#3	2.281(4)
O(009)-C(00C)	1.38(2)

O(00A)-C(00H)	1.254(12)
C(00C)-C(5)	1.57(2)
C(00E)-C(00H)	1.52(3)
C(00E)-C(00K)#4	1.30(2)
C(00E)-C(00K)	1.30(2)
C(00H)-O(00A)#4	1.254(12)
C(00I)-H(00A)	0.9600
C(00I)-H(00B)	0.9600
C(00I)-H(00C)	0.9600
C(00I)-C(5)	1.48(2)
C(00K)-H(00D)	0.9600
C(00K)-H(00E)	0.9600
C(00K)-H(00F)	0.9600
C(00N)-O(005)#5	1.435(16)
C(00N)-C(6)	1.38(4)
C(5)-H(5)	0.9800
C(5)-C(46)	1.47(2)
C(6)-C(12)#5	1.47(2)
C(6)-C(12)	1.47(2)
C(6)-C(31)#5	1.53(3)
C(6)-C(31)	1.53(2)
C(12)-C(31)	0.68(6)
O(14)-C(22)	1.14(2)
O(17)-C(22)	1.37(2)
C(22)-C(37)	1.40(2)
C(37)-H(37)	0.9800
C(37)-C(45)	1.50(2)
C(37)-C(20)	1.47(2)
C(45)-H(45A)	0.9600
C(45)-H(45B)	0.9600
C(45)-H(45C)	0.9600
C(20)-H(20A)	0.9600
C(20)-H(20B)	0.9600
C(20)-H(20C)	0.9600
C(46)-H(46A)	0.9600
C(46)-H(46B)	0.9600

C(46)-H(46C) 0.9600

Zr(02)#1-Zr(01)-Zr(02) 60.48(4)
O(004)#1-Zr(01)-Zr(02)#1 32.61(19)
O(004)-Zr(01)-Zr(02) 32.61(19)
O(004)-Zr(01)-Zr(02)#1 83.3(2)
O(004)#1-Zr(01)-Zr(02) 83.3(2)
O(004)#1-Zr(01)-O(004) 92.3(5)
O(004)-Zr(01)-O(005) 143.4(4)
O(004)#1-Zr(01)-O(005) 85.2(5)
O(004)#1-Zr(01)-O(005)#2143.4(4)
O(004)-Zr(01)-O(005)#2 85.2(5)
O(004)-Zr(01)-O(007) 71.1(3)
O(004)#1-Zr(01)-O(007) 71.1(3)
O(004)-Zr(01)-O(008) 69.6(2)
O(004)#1-Zr(01)-O(008) 69.6(2)
O(004)#1-Zr(01)-O(009)#276.3(8)
O(004)-Zr(01)-O(009) 76.3(8)
O(004)#1-Zr(01)-O(009) 146.4(7)
O(004)-Zr(01)-O(009)#2 146.4(7)
O(004)-Zr(01)-O(14)#2 139.1(5)
O(004)-Zr(01)-O(14) 86.8(7)
O(004)#1-Zr(01)-O(14)#2 86.8(7)
O(004)#1-Zr(01)-O(14) 139.1(5)
O(005)#2-Zr(01)-Zr(02) 111.0(4)
O(005)-Zr(01)-Zr(02) 167.4(4)
O(005)-Zr(01)-Zr(02)#1 111.0(4)
O(005)#2-Zr(01)-Zr(02)#1167.4(5)
O(005)-Zr(01)-O(005)#2 75.7(7)
O(005)#2-Zr(01)-O(007) 140.2(3)
O(005)-Zr(01)-O(007) 140.2(3)
O(005)-Zr(01)-O(008) 75.4(4)
O(005)#2-Zr(01)-O(008) 75.4(4)
O(005)#2-Zr(01)-O(009)#2122.5(9)
O(005)-Zr(01)-O(009) 122.5(9)
O(005)#2-Zr(01)-O(009) 68.2(6)

O(005)-Zr(01)-O(009)#2	68.2(6)
O(005)-Zr(01)-O(14)#2	77.3(6)
O(005)-Zr(01)-O(14)	118.1(8)
O(005)#2-Zr(01)-O(14)#2	118.1(8)
O(005)#2-Zr(01)-O(14)	77.3(6)
O(007)-Zr(01)-Zr(02)#1	38.68(13)
O(007)-Zr(01)-Zr(02)	38.68(13)
O(008)-Zr(01)-Zr(02)	95.70(19)
O(008)-Zr(01)-Zr(02)#1	95.70(19)
O(008)-Zr(01)-O(007)	122.0(3)
O(008)-Zr(01)-O(009)#2	131.5(6)
O(008)-Zr(01)-O(009)	131.5(6)
O(009)#2-Zr(01)-Zr(02)#1	70.0(8)
O(009)#2-Zr(01)-Zr(02)	113.8(6)
O(009)-Zr(01)-Zr(02)#1	113.8(6)
O(009)-Zr(01)-Zr(02)	70.0(8)
O(009)-Zr(01)-O(007)	75.2(6)
O(009)#2-Zr(01)-O(007)	75.2(6)
O(009)#2-Zr(01)-O(009)	95.6(13)
O(14)-Zr(01)-Zr(02)#1	107.2(5)
O(14)#2-Zr(01)-Zr(02)#1	74.2(6)
O(14)-Zr(01)-Zr(02)	74.2(6)
O(14)#2-Zr(01)-Zr(02)	107.2(5)
O(14)#2-Zr(01)-O(007)	70.0(6)
O(14)-Zr(01)-O(007)	70.0(6)
O(14)-Zr(01)-O(008)	145.1(5)
O(14)#2-Zr(01)-O(008)	145.1(5)
O(14)#2-Zr(01)-O(14)	68.5(11)
O(003)-Zr(02)-Zr(01)#3	86.0(3)
O(003)-Zr(02)-O(006)	153.0(6)
O(003)-Zr(02)-O(006)#4	153.0(6)
O(003)-Zr(02)-O(007)	70.3(2)
O(003)-Zr(02)-O(007)#3	70.3(2)
O(003)-Zr(02)-O(00A)	87.8(4)
O(003)-Zr(02)-O(00A)#4	87.8(4)
O(003)-Zr(02)-C(00H)	87.3(7)

O(003)-Zr(02)-O(17)#4	137.7(5)
O(003)-Zr(02)-O(17)	137.7(5)
O(004)-Zr(02)-Zr(01)#3	32.20(7)
O(004)-Zr(02)-O(003)	96.1(4)
O(004)-Zr(02)-O(006)#4	82.0(6)
O(004)-Zr(02)-O(006)	82.0(6)
O(004)-Zr(02)-O(007)	74.4(3)
O(004)-Zr(02)-O(007)#3	74.4(3)
O(004)-Zr(02)-O(00A)#4	151.4(3)
O(004)-Zr(02)-O(00A)	151.4(3)
O(004)-Zr(02)-C(00H)	176.6(7)
O(004)-Zr(02)-O(17)	84.7(6)
O(004)-Zr(02)-O(17)#4	84.7(6)
O(006)-Zr(02)-Zr(01)#3	104.7(5)
O(006)#4-Zr(02)-Zr(01)#3	78.2(6)
O(006)#4-Zr(02)-O(006)	53.6(11)
O(006)#4-Zr(02)-O(007)#3	83.5(6)
O(006)-Zr(02)-O(007)#3	133.6(5)
O(006)-Zr(02)-O(007)	83.5(6)
O(006)#4-Zr(02)-O(007)	133.6(5)
O(006)-Zr(02)-O(00A)	81.9(6)
O(006)#4-Zr(02)-O(00A)#4	81.9(6)
O(006)-Zr(02)-O(00A)#4	106.7(7)
O(006)#4-Zr(02)-O(00A)	106.7(7)
O(006)#4-Zr(02)-C(00H)	94.9(8)
O(006)-Zr(02)-C(00H)	94.9(8)
O(007)-Zr(02)-Zr(01)#3	99.5(2)
O(007)#3-Zr(02)-Zr(01)#3	42.3(2)
O(007)#3-Zr(02)-O(007)	125.7(4)
O(007)#3-Zr(02)-O(00A)	132.9(4)
O(007)-Zr(02)-O(00A)#4	132.9(4)
O(007)-Zr(02)-O(00A)	80.4(3)
O(007)#3-Zr(02)-O(00A)#4	80.4(3)
O(007)-Zr(02)-C(00H)	106.9(4)
O(007)#3-Zr(02)-C(00H)	106.9(4)
O(007)#3-Zr(02)-O(17)#4	69.4(6)

O(007)-Zr(02)-O(17)#4	147.6(5)
O(007)#3-Zr(02)-O(17)	147.6(5)
O(007)-Zr(02)-O(17)	69.4(6)
O(00A)-Zr(02)-Zr(01)#3	173.4(2)
O(00A)#4-Zr(02)-Zr(01)#3	120.7(3)
O(00A)#4-Zr(02)-O(00A)	56.8(5)
O(00A)-Zr(02)-C(00H)	28.4(3)
O(00A)#4-Zr(02)-C(00H)	28.4(3)
C(00H)-Zr(02)-Zr(01)#3	148.68(13)
O(17)#4-Zr(02)-Zr(01)#3	72.4(6)
O(17)-Zr(02)-Zr(01)#3	112.4(5)
O(17)-Zr(02)-O(00A)#4	111.2(6)
O(17)#4-Zr(02)-O(00A)	111.2(6)
O(17)#4-Zr(02)-O(00A)#4	73.8(5)
O(17)-Zr(02)-O(00A)	73.8(5)
O(17)-Zr(02)-C(00H)	92.8(7)
O(17)#4-Zr(02)-C(00H)	92.8(7)
O(17)#4-Zr(02)-O(17)	84.5(10)
Zr(02)-O(003)-Zr(02)#3	113.6(2)
Zr(02)#1-O(003)-Zr(02)#3	113.6(2)
Zr(02)-O(003)-Zr(02)#1	113.6(2)
Zr(01)-O(004)-Zr(01)#3	119.2(3)
Zr(01)-O(004)-Zr(02)	115.2(2)
Zr(01)#3-O(004)-Zr(02)	115.2(2)
C(00N)-O(005)-Zr(01)	126.2(15)
C(00C)-O(006)-Zr(02)	129(2)
Zr(02)#1-O(007)-Zr(01)	99.0(3)
Zr(02)-O(007)-Zr(01)	99.0(3)
Zr(02)#1-O(007)-Zr(02)	105.5(4)
Zr(01)-O(008)-Zr(01)#3	101.5(3)
Zr(01)-O(008)-Zr(01)#1	101.5(3)
Zr(01)#3-O(008)-Zr(01)#1	101.5(3)
C(00C)-O(009)-Zr(01)	130(2)
C(00H)-O(00A)-Zr(02)	91.7(9)
O(006)-C(00C)-O(009)	129(2)
O(006)-C(00C)-C(5)	121(2)

O(009)-C(00C)-C(5)	108(2)
C(00K)#4-C(00E)-C(00H)	124.3(15)
C(00K)-C(00E)-C(00H)	124.3(15)
C(00K)#4-C(00E)-C(00K)	110(3)
O(00A)#4-C(00H)-Zr(02)	59.9(8)
O(00A)-C(00H)-Zr(02)	59.9(8)
O(00A)#4-C(00H)-O(00A)	119.8(17)
O(00A)-C(00H)-C(00E)	120.1(8)
O(00A)#4-C(00H)-C(00E)	120.1(8)
C(00E)-C(00H)-Zr(02)	178(2)
H(00A)-C(00I)-H(00B)	109.5
H(00A)-C(00I)-H(00C)	109.5
H(00B)-C(00I)-H(00C)	109.5
C(5)-C(00I)-H(00A)	109.5
C(5)-C(00I)-H(00B)	109.5
C(5)-C(00I)-H(00C)	109.5
C(00E)-C(00K)-H(00D)	109.5
C(00E)-C(00K)-H(00E)	109.5
C(00E)-C(00K)-H(00F)	109.5
H(00D)-C(00K)-H(00E)	109.5
H(00D)-C(00K)-H(00F)	109.5
H(00E)-C(00K)-H(00F)	109.5
O(005)-C(00N)-O(005)#5	132(2)
C(6)-C(00N)-O(005)	113.7(12)
C(6)-C(00N)-O(005)#5	113.7(12)
C(00C)-C(5)-H(5)	92.6
C(00I)-C(5)-C(00C)	119(2)
C(00I)-C(5)-H(5)	92.6
C(46)-C(5)-C(00C)	118(2)
C(46)-C(5)-C(00I)	122(2)
C(46)-C(5)-H(5)	92.6
C(00N)-C(6)-C(12)#5	131(2)
C(00N)-C(6)-C(12)	131(2)
C(00N)-C(6)-C(31)#5	108.6(19)
C(00N)-C(6)-C(31)	108.6(19)
C(12)#5-C(6)-C(12)	91(3)

C(12)#5-C(6)-C(31)#5	26(2)
C(12)-C(6)-C(31)	26(2)
C(12)-C(6)-C(31)#5	117(2)
C(12)#5-C(6)-C(31)	117(2)
C(31)-C(6)-C(31)#5	142(3)
C(31)-C(12)-C(6)	82(3)
C(22)-O(14)-Zr(01)	140(2)
C(22)-O(17)-Zr(02)	130.9(16)
O(14)-C(22)-O(17)	123(2)
O(14)-C(22)-C(37)	120(2)
O(17)-C(22)-C(37)	117(2)
C(22)-C(37)-H(37)	91.0
C(22)-C(37)-C(45)	118(2)
C(22)-C(37)-C(20)	121(2)
C(45)-C(37)-H(37)	91.0
C(20)-C(37)-H(37)	91.0
C(20)-C(37)-C(45)	121.2(19)
C(37)-C(45)-H(45A)	109.5
C(37)-C(45)-H(45B)	109.5
C(37)-C(45)-H(45C)	109.5
H(45A)-C(45)-H(45B)	109.5
H(45A)-C(45)-H(45C)	109.5
H(45B)-C(45)-H(45C)	109.5
C(37)-C(20)-H(20A)	109.5
C(37)-C(20)-H(20B)	109.5
C(37)-C(20)-H(20C)	109.5
H(20A)-C(20)-H(20B)	109.5
H(20A)-C(20)-H(20C)	109.5
H(20B)-C(20)-H(20C)	109.5
C(5)-C(46)-H(46A)	109.5
C(5)-C(46)-H(46B)	109.5
C(5)-C(46)-H(46C)	109.5
H(46A)-C(46)-H(46B)	109.5
H(46A)-C(46)-H(46C)	109.5
H(46B)-C(46)-H(46C)	109.5
C(12)-C(31)-C(6)	72(3)

Symmetry transformations used to generate equivalent atoms:

#1 -y+1,x-y,z #2 -y+1,-x+1,z #3 -x+y+1,-x+1,z

#4 -x+y+1,y,z #5 x,x-y,z

Supplementary Table 4 | Anisotropic displacement parameters (Å$^2 \times 10^3$) for Zr-MOC (CCDC No: 2022033). The anisotropic displacement factor exponent takes the form: -2p^2[h^2 a*^{2}U^{11} + ... + 2 h k a* b* U^{12}]

	U^{11}	U^{22}	U^{33}	U^{23}	U^{13}	U^{12}
Zr(01)	87(1)	87(1)	36(1)	2(1)	-2(1)	58(1)
Zr(02)	96(1)	65(1)	33(1)	-10(1)	-5(1)	33(1)
O(003)	59(4)	59(4)	44(6)	0	0	29(2)
O(004)	76(4)	65(4)	39(4)	-15(3)	-7(2)	33(2)
O(005)	117(6)	317(16)	65(5)	-14(7)	-32(5)	105(8)
O(006)	113(13)	90(11)	55(8)	-1(7)	15(9)	59(12)
O(007)	107(6)	107(6)	56(5)	-2(2)	2(2)	73(6)
O(008)	77(5)	77(5)	23(5)	0	0	39(3)
O(009)	138(15)	94(12)	87(11)	23(9)	49(10)	71(11)
O(00A)	167(7)	123(6)	51(4)	-23(4)	7(4)	80(6)
C(00C)	125(12)	82(10)	81(9)	-2(8)	32(10)	68(10)
C(00E)	224(15)	209(14)	93(10)	-26(12)	-13(6)	105(7)
C(00H)	188(11)	168(10)	56(8)	-16(9)	-8(4)	84(5)
C(00I)	125(18)	101(16)	170(20)	8(16)	-7(18)	73(15)
C(00K)	280(20)	280(20)	108(13)	-44(16)	2(14)	109(18)
C(00N)	138(11)	325(19)	85(10)	-13(4)	-25(9)	69(6)
C(5)	136(14)	84(12)	106(12)	-5(10)	21(12)	59(12)
C(6)	145(12)	340(20)	105(12)	-10(5)	-20(10)	72(6)
C(12)	153(15)	350(20)	115(16)	1(12)	-21(14)	69(12)
O(14)	118(13)	90(11)	62(8)	27(7)	39(9)	74(10)
O(17)	109(12)	69(9)	62(8)	8(6)	41(9)	43(10)
C(22)	117(12)	71(9)	74(8)	15(7)	17(9)	65(9)
C(37)	136(14)	88(11)	104(11)	13(10)	8(12)	56(11)
C(45)	140(20)	69(14)	150(20)	0(14)	0(20)	39(16)
C(20)	160(20)	95(15)	180(20)	-4(17)	0(20)	81(15)
C(46)	140(20)	130(20)	130(20)	-4(17)	17(19)	48(18)
C(31)	150(15)	350(20)	112(16)	-4(12)	-26(14)	67(12)

**Supplementary Table 5 | ** Hydrogen coordinates ($\times 10^4$) and isotropic displacement parameters ($Å^2 \times 10^3$) for Zr-MOC (CCDC No: 2022033).

	x	y	z	U(eq)
H(00A)	7186	-610	4492	188
H(00B)	6454	-823	5092	188
H(00C)	7218	163	4965	188
H(00D)	6865	1630	987	356
H(00E)	6458	1838	294	356
H(00F)	6133	847	515	356
H(5)	6701	-177	3774	128
H(37)	7248	188	3817	131
H(45A)	5739	-593	3579	192
H(45B)	5791	-1232	4164	192
H(45C)	6404	-948	3463	192
H(20A)	8023	500	4725	205
H(20B)	7554	-523	4553	205
H(20C)	7227	-180	5216	205
H(46A)	5108	-880	3634	207
H(46B)	5268	-1541	4113	207
H(46C)	5771	-1187	3361	207

Supplementary Table 6 | Atomistic coordinates of $Li(EC)_4^+$.

Atom	*x (Å)*	*y (Å)*	*z (Å)*
C	0.81055	-0.17651	-2.82520
O	1.16210	0.55404	-1.90386
O	1.66046	-0.96171	-3.51380
O	-0.45858	-0.27170	-3.26931
C	0.93646	-1.53404	-4.65305
C	-0.52488	-1.36973	-4.23698
H	1.25044	-2.57958	-4.76799
H	-1.18705	-1.06412	-5.05736
H	1.20185	-0.94712	-5.54624
H	-0.93080	-2.25132	-3.71677
O	-0.91583	2.72220	-0.72555
C	-1.69605	3.38162	-0.04446
O	-2.31052	4.49196	-0.49354
O	-2.03185	3.07868	1.22471
C	-3.00177	5.12056	0.63750
C	-3.12386	3.96916	1.63354
H	-2.36601	5.94315	1.00026
H	-4.06563	3.40712	1.53501
H	-3.96625	5.50261	0.27945
H	-2.95319	4.26395	2.67686
Li	0.11066	1.11667	-0.35175
O	-0.97488	-0.40781	0.26711
O	-1.11759	-2.45374	1.23289
C	-0.44877	-3.74165	1.02737
C	-0.00625	-3.66154	-0.43341
O	0.03150	-2.21741	-0.67735
C	-0.70172	-1.60342	0.27433
H	-1.17412	-4.54060	1.22756
H	0.39504	-3.79612	1.73312
H	0.99803	-4.06570	-0.61526
H	-0.73530	-4.09963	-1.13279
O	1.36339	1.42093	1.13725
C	1.86639	0.60539	1.90320
O	1.82016	-0.73208	1.72089
C	2.33584	-1.37753	2.93204
C	3.14909	-0.25850	3.57980
O	2.53046	0.94944	3.02252
H	1.47066	-1.69022	3.53734
H	2.93625	-2.24499	2.62908
H	3.05543	-0.21706	4.67247
H	4.20785	-0.26026	3.27767

Supplementary Table 7 | Atomistic coordinates of $Li(EC)_3^+$.

Atom	*x (Å)*	*y (Å)*	*z (Å)*
O	-0.21906	1.10537	0.29851
O	-2.15328	1.66473	1.33224
O	2.63218	-2.56352	-2.85835
O	1.56289	-0.90441	-1.74903
O	2.93686	0.30279	0.83686
O	1.37906	-3.06332	-1.07000
C	-1.09841	0.86323	1.12533
C	-2.88059	1.16787	2.50820
H	-3.95540	1.25656	2.30551
H	-2.58978	1.79490	3.36503
C	5.44460	-2.03455	0.32272
H	5.95804	-1.77164	-0.61492
H	5.43393	-3.12159	0.47312
C	1.84222	-2.09576	-1.87898
C	3.94181	-0.40715	0.86781
C	5.95055	-1.24993	1.53225
H	5.83267	-1.79247	2.48288
H	6.97512	-0.87322	1.42107
C	-2.38930	-0.27458	2.61972
H	-2.19956	-0.60053	3.65020
H	-3.03087	-0.99633	2.09116
C	2.89704	-3.98303	-2.59396
H	3.89782	-4.05443	-2.13992
H	2.86460	-4.52030	-3.55019
C	1.76845	-4.36122	-1.63489
H	0.88604	-4.77810	-2.14398
H	2.08629	-5.01096	-0.80957
Li	1.35850	0.20608	-0.23014
O	-1.10266	-0.22748	1.91165
O	4.05416	-1.57585	0.20828
O	5.04190	-0.09561	1.56868

Supplementary Table 8 | Atomistic coordinates of $Li(EC)_2^+$.

Atom	*x (Å)*	*y (Å)*	*z (Å)*
O	2.93553	1.26392	-0.27020
O	3.46196	2.62468	1.45475
O	4.15930	4.73961	4.40055
O	1.30983	2.40212	0.76674
C	6.82858	4.09750	6.51363
H	7.90404	4.07287	6.29782
H	6.53909	3.26253	7.16992
C	2.62146	2.13058	0.70044
C	5.04477	4.74976	5.25881
C	6.32552	5.45521	7.00063
H	6.13101	5.49657	8.07968
H	6.96035	6.29640	6.68263
C	1.68252	0.73856	-0.83303
H	1.50975	-0.25053	-0.38198
H	1.80663	0.65730	-1.92020
C	0.65972	1.78769	-0.40189
H	0.50719	2.58227	-1.14803
H	-0.29912	1.36605	-0.07519
Li	3.67180	3.76477	2.89709
O	6.10524	3.92835	5.24366
O	5.03843	5.58886	6.30147

**Supplementary Table 9 | ** Atomistic coordinates of ethylene carbonate (EC, $C_3H_4O_3$).

**Atom**	***x (Å)***	***y (Å)***	***z (Å)***
O	1.33463	1.32465	1.46387
C	2.17543	0.77304	2.15053
O	2.46859	-0.55406	2.08397
C	3.36792	-0.88448	3.18748
C	3.96443	0.47437	3.54874
O	2.94010	1.40602	3.08119
H	2.76379	-1.31939	4.00034
H	4.10727	-1.61166	2.82625
H	4.10365	0.62504	4.62775
H	4.89772	0.69378	3.00474

Supplementary Table 10 | Atomistic coordinates of Li(EC)$_4$.

Atom	*x (Å)*	*y (Å)*	*z (Å)*
C	1.63838	0.14729	-0.07796
O	1.96170	0.89255	0.83803
O	2.51182	-0.64447	-0.73731
O	0.38100	0.03374	-0.55710
C	1.81846	-1.24265	-1.87965
C	0.34769	-1.08768	-1.49548
H	2.14550	-2.28668	-1.97180
H	-0.30337	-0.81373	-2.33581
H	2.09352	-0.66861	-2.77865
H	-0.05489	-1.96218	-0.96046
O	-0.10175	3.06255	2.09288
C	-0.62437	3.94664	2.85445
O	-1.33124	5.02492	2.20622
O	-1.52589	3.47606	3.90938
C	-2.06999	5.66367	3.25463
C	-2.52513	4.48976	4.13422
H	-1.41335	6.35630	3.81951
H	-3.51233	4.10010	3.82089
H	-2.90334	6.22756	2.80813
H	-2.56812	4.75442	5.20592
Li	0.84194	1.50853	2.39636
O	-0.11251	-0.17773	2.99129
O	-0.14256	-2.25468	3.90453
C	0.51185	-3.52654	3.59528
C	0.81115	-3.39664	2.10194
O	0.81880	-1.94769	1.90132
C	0.17318	-1.36571	2.93722
H	-0.18500	-4.33944	3.83719
H	1.42016	-3.59829	4.21438
H	1.79458	-3.78878	1.81106
H	0.01955	-3.82070	1.46422
O	2.21052	1.61507	3.87276
C	2.77882	0.75550	4.53208
O	2.76663	-0.56590	4.23837
C	3.35743	-1.28894	5.36628
C	4.18436	-0.20478	6.05262
O	3.49703	1.02277	5.64433
H	2.53341	-1.66389	5.99413
H	3.95481	-2.11919	4.96809
H	4.16689	-0.25997	7.14866
H	5.21975	-0.14962	5.68092

Supplementary Table 11 | Atomistic coordinates of Li(EC)$_4$-TS.

Atom	x (Å)	y (Å)	z (Å)
C	0.74781	-0.08556	-2.60047
O	1.11364	0.61194	-1.66325
O	1.58106	-0.87896	-3.30877
O	-0.52211	-0.13959	-3.05656
C	0.84561	-1.40331	-4.46134
C	-0.61082	-1.22040	-4.03829
H	1.13739	-2.45149	-4.60791
H	-1.27079	-0.89278	-4.85214
H	1.11992	-0.79729	-5.33937
H	-1.02909	-2.10354	-3.53003
O	-0.90339	2.83146	-0.46115
C	-1.42464	3.71149	0.31423
O	-1.94252	4.86999	-0.28122
O	-2.08803	3.43443	1.45724
C	-3.19573	5.26707	0.41683
C	-3.63246	4.08053	1.22022
H	-2.97375	6.14232	1.05289
H	-4.21899	3.32421	0.68021
H	-3.90089	5.55152	-0.37999
H	-3.98499	4.26005	2.24100
Li	0.00767	1.25503	-0.10584
O	-0.97523	-0.41880	0.49088
O	-1.00243	-2.50357	1.38642
C	-0.36561	-3.77821	1.05271
C	-0.09373	-3.63719	-0.44499
O	-0.08212	-2.18674	-0.63432
C	-0.70002	-1.60877	0.42033
H	-1.06518	-4.58703	1.30037
H	0.55357	-3.86405	1.65367
H	0.88210	-4.03174	-0.75736
H	-0.89909	-4.05175	-1.07157
O	1.39751	1.34005	1.35037
C	1.96907	0.46693	1.98902
O	1.92880	-0.85195	1.68678
C	2.53738	-1.59228	2.79403
C	3.39592	-0.52525	3.46866
O	2.71765	0.71531	3.08552
H	1.72412	-1.96200	3.43876
H	3.11379	-2.42681	2.37444
H	3.40312	-0.58822	4.56441
H	4.42313	-0.48253	3.07319

Supplementary Table 12 | Atomistic coordinates of $H_2\dot{C}CH_2OCO_2Li(EC)_3$.

Atom	*x (Å)*	*y (Å)*	*z (Å)*
C	0.58295	-0.15207	0.01987
O	0.96958	0.53988	0.95290
O	1.40405	-0.92234	-0.72701
O	-0.70082	-0.22189	-0.39284
C	0.63901	-1.44172	-1.86236
C	-0.80553	-1.28727	-1.38945
H	0.94119	-2.48319	-2.03343
H	-1.49609	-0.95608	-2.17601
H	0.87559	-0.81956	-2.73999
H	-1.19494	-2.18486	-0.88367
O	-1.04402	2.70101	2.23008
C	-1.79270	3.36144	3.02270
O	-2.28003	4.54043	2.40775
O	-2.11629	3.11257	4.20797
C	-3.21341	5.31924	3.19873
C	-4.56869	4.71480	3.25235
H	-2.81012	5.48158	4.21353
H	-5.00045	4.26405	2.35267
H	-3.23056	6.30016	2.67858
H	-5.16784	4.74438	4.16671
Li	-0.08947	1.13671	2.56261
O	-1.04129	-0.54114	3.15457
O	-1.04160	-2.63351	4.03092
C	-0.39851	-3.89997	3.67906
C	-0.14248	-3.74308	2.17995
O	-0.14634	-2.29100	2.00334
C	-0.75759	-1.72817	3.06957
H	-1.08944	-4.71642	3.92599
H	0.52704	-3.98417	4.27034
H	0.83395	-4.12550	1.85469
H	-0.94997	-4.15963	1.55746
O	1.35992	1.22968	3.95833
C	1.93355	0.35652	4.59514
O	1.89040	-0.96220	4.29337
C	2.50371	-1.70271	5.39794
C	3.36799	-0.63661	6.06677
O	2.68981	0.60490	5.68592
H	1.69324	-2.07026	6.04742
H	3.07629	-2.53862	4.97594
H	3.38091	-0.69838	7.16253
H	4.39307	-0.59603	5.66567

Supplementary Table 13 | Atomistic coordinates of $(EC)_3LiO_2CO(CH_2)_4OCO_2Li(EC)_3$.

Atom	*x (Å)*	*y (Å)*	*z (Å)*
C	0.97142	-0.89370	0.45166
O	1.04591	0.26866	0.82864
O	2.02973	-1.73297	0.41409
O	-0.16270	-1.47959	0.01133
C	1.61663	-2.96144	-0.26686
C	0.09441	-2.91034	-0.14986
H	2.07393	-3.81333	0.25304
H	-0.43133	-3.25540	-1.04956
H	1.97191	-2.90516	-1.30798
H	-0.28937	-3.42447	0.74549
O	-1.49824	2.18400	0.36085
C	-2.53725	2.90783	0.51614
O	-3.21815	3.09944	-0.70767
O	-2.98512	3.44215	1.55832
C	-4.46757	3.81778	-0.64560
C	-5.64098	2.90859	-0.28242
H	-4.38332	4.64798	0.07713
H	-5.65777	2.05102	-0.98174
H	-4.60663	4.23676	-1.65816
H	-5.47428	2.50172	0.73157
Li	-0.41411	1.39415	1.64733
O	-1.26886	0.17311	3.01123
O	-1.19735	-1.03831	4.92832
C	-0.34520	-2.11408	5.43612
C	0.18270	-2.75748	4.15436
O	0.04955	-1.67722	3.17686
C	-0.83291	-0.77091	3.65544
H	-0.96468	-2.78471	6.04555
H	0.44787	-1.65598	6.04810
H	1.24091	-3.04543	4.20645
H	-0.43404	-3.60213	3.80854
O	0.67688	2.55510	2.88216
C	1.17064	2.34411	3.98144
O	1.32879	1.11462	4.52494
C	1.74333	1.28145	5.91944
C	2.31511	2.69738	5.92502
O	1.62663	3.32211	4.79318
H	0.84724	1.17642	6.55143
H	2.48140	0.50381	6.15444
H	2.07504	3.26898	6.83085
H	3.39659	2.72837	5.71872

C	-13.27990	7.87933	-0.42769
O	-13.25358	7.09797	0.51458
O	-14.28218	8.76067	-0.63570
O	-12.32907	7.94908	-1.38394
C	-14.07717	9.39383	-1.93935
C	-12.59899	9.12027	-2.21673
H	-14.32032	10.46044	-1.84549
H	-12.38657	8.85632	-3.26085
H	-14.75033	8.90450	-2.66080
H	-11.93640	9.93287	-1.88004
O	-10.94004	4.83571	0.66023
C	-9.85976	4.22048	0.94763
O	-9.42785	3.40945	-0.12680
O	-9.19133	4.24198	2.00808
C	-8.15668	2.74695	0.03515
C	-6.98283	3.65130	-0.33861
H	-8.05391	2.38867	1.07426
H	-7.14905	4.04770	-1.35843
H	-8.20011	1.87529	-0.64201
H	-6.96748	4.51545	0.35026
Li	-11.63031	6.36319	1.46508
O	-10.39061	7.94590	1.61340
O	-9.76706	9.97383	2.41610
C	-10.38590	11.29890	2.46636
C	-11.37702	11.25233	1.30331
O	-11.59412	9.81692	1.12478
C	-10.57557	9.15063	1.71408
H	-9.59616	12.05168	2.34457
H	-10.87478	11.40563	3.44762
H	-12.34463	11.72114	1.52620
H	-10.96001	11.64916	0.36428
O	-12.32580	6.25688	3.35009
C	-12.41241	7.09033	4.24162
O	-12.30827	8.42600	4.05076
C	-12.24038	9.07189	5.36277
C	-12.85575	8.01763	6.28057
O	-12.62847	6.77622	5.53695
H	-11.17977	9.27109	5.58378
H	-12.81280	10.00730	5.31596
H	-12.35583	7.93101	7.25390
H	-13.94297	8.13754	6.40974

Supplementary Table 14 | Atomistic coordinates of $(EC)_3LiO_2CO(CH_2)_2OCO_2Li(EC)_3$.

Atom	*x (Å)*	*y (Å)*	*z (Å)*
C	-0.46108	-0.64124	3.06053
O	-1.29180	0.03442	2.47246
O	0.68003	-0.15331	3.59683
O	-0.59360	-1.97303	3.27242
C	1.50418	-1.27757	4.03171
C	0.48713	-2.41284	4.15277
H	1.98024	-1.00769	4.98361
H	0.85807	-3.37935	3.78647
H	2.26481	-1.45850	3.25620
H	0.07525	-2.51698	5.16924
O	-1.02105	3.23012	2.51854
C	-1.04990	4.05713	1.54943
O	-1.01117	5.42006	1.81574
O	-1.10895	3.74935	0.32220
Li	-1.06453	1.76890	1.12063
O	0.55477	0.95784	0.16141
O	1.11751	-0.51161	-1.47481
C	1.33514	-1.95570	-1.53432
C	1.69601	-2.30556	-0.08883
O	1.14270	-1.18292	0.66479
C	0.90196	-0.16043	-0.18811
H	2.14441	-2.15211	-2.24969
H	0.39836	-2.42414	-1.87422
H	1.22503	-3.22889	0.27427
H	2.78215	-2.32919	0.09243
O	-2.66670	0.88830	0.10261
C	-2.83126	-0.32369	0.10309
O	-1.91909	-1.21100	-0.35756
C	-2.50874	-2.54707	-0.34443
C	-3.72483	-2.37480	0.56951
O	-3.95615	-0.93170	0.53810
H	-2.77728	-2.80722	-1.38027
H	-1.76119	-3.24901	0.04914
H	-4.62871	-2.87642	0.19947
H	-3.52450	-2.65932	1.61399
C	-6.03308	11.58481	0.61030
O	-5.61218	11.69841	1.75442
O	-7.18533	12.14566	0.18153
O	-5.41591	10.87722	-0.35970
C	-7.46787	11.64157	-1.16370
C	-6.10109	11.13795	-1.62489

H	-7.86203	12.47074	-1.76559
H	-6.14280	10.19968	-2.19325
H	-8.21578	10.83809	-1.07181
H	-5.52073	11.89769	-2.17189
O	-3.16652	9.80108	2.61639
C	-2.03780	9.33050	2.97814
O	-2.02168	7.92132	2.92668
O	-1.00435	9.93546	3.35316
C	-0.78467	7.29624	3.30954
C	-0.93385	5.79428	3.20868
H	0.04081	7.64862	2.66480
H	-1.83839	5.45268	3.74303
H	-0.53218	7.55709	4.35430
H	-0.05648	5.31399	3.67741
Li	-3.68760	11.56511	2.34929
O	-2.69249	12.71390	1.01737
O	-2.20404	14.70486	0.04571
C	-2.98531	15.74703	-0.62095
C	-4.21690	14.98470	-1.11014
O	-4.24898	13.82350	-0.22147
C	-3.02740	13.67462	0.33848
H	-2.37586	16.16817	-1.43096
H	-3.22336	16.52020	0.12648
H	-5.15779	15.53714	-0.98723
H	-4.11487	14.61535	-2.14279
O	-3.71243	12.81852	3.92929
C	-3.73398	14.04053	3.98748
O	-3.96279	14.85187	2.92881
C	-3.68920	16.22959	3.34138
C	-3.81745	16.14817	4.86062
O	-3.53253	14.73615	5.12750
H	-2.67071	16.48439	3.00740
H	-4.43165	16.88433	2.86711
H	-3.08042	16.75557	5.40156
H	-4.83697	16.35852	5.22052

Supplementary Table 15 | Atomistic coordinates of C_2H_4.

Atom	*x (Å)*	*y (Å)*	*z (Å)*
C	-4.80678	3.60065	3.53352
C	-6.08852	3.49182	3.14662
H	-4.05901	2.84467	3.26701
H	-6.83629	4.24779	3.41314
H	-4.45631	4.44979	4.13173
H	-6.43898	2.64268	2.54841

Supplementary Table 16 | Atomistic coordinates of $[(EC)_3Li(CO_3)]^-$.

Atom	*x (Å)*	*y (Å)*	*z (Å)*
C	0.81055	-0.17651	-2.8252
C	1.02922	-1.50695	0.92002
O	1.24746	-2.55537	1.50743
O	0.01998	-1.33571	0.02964
O	1.77056	-0.38246	1.06376
C	0.16226	-0.02449	-0.60106
C	1.12235	0.71416	0.33587
H	-0.83202	0.43905	-0.65714
H	1.90663	1.26910	-0.19735
H	0.57020	-0.18317	-1.61223
H	0.60525	1.37356	1.05467
O	-0.66892	2.94753	1.71892
C	-0.80013	4.14036	2.29625
O	-1.08601	5.19243	1.61969
O	-0.61573	4.16906	3.60860
Li	-0.25036	2.30847	3.47468
O	-1.29139	0.91049	4.50438
O	-1.71062	-1.22978	5.13228
C	-1.65456	-2.52902	4.46217
C	-1.82406	-2.15834	2.98908
O	-1.40405	-0.75749	2.96003
C	-1.44936	-0.26899	4.21816
H	-2.46567	-3.15500	4.85673
H	-0.67485	-2.98077	4.68465
H	-1.16965	-2.72534	2.31470
H	-2.87083	-2.20125	2.64934
O	1.63396	1.60250	3.73837
C	2.15616	0.50745	3.89856
O	1.48577	-0.60938	4.25720
C	2.44755	-1.68318	4.49389
C	3.71880	-1.15538	3.82507
O	3.48099	0.28570	3.75936
H	2.54878	-1.80814	5.58347
H	2.05804	-2.59552	4.02482
H	4.63030	-1.32558	4.41310
H	3.84552	-1.52122	2.79485

Supplementary Table 17 | Atomistic coordinates of $(EC)_4Li(CH_2)_2OCO_2Li(EC)_3$.

Atom	x (Å)	y (Å)	z (Å)
C	-1.68502	0.40963	-0.01254
O	-2.22431	0.74641	-1.05461
O	-1.01729	1.26726	0.79825
O	-1.66493	-0.85680	0.46461
C	-0.33710	0.48677	1.83067
C	-1.10882	-0.83149	1.81450
H	-0.41045	1.03239	2.78065
H	-0.47391	-1.71796	1.94289
H	0.71682	0.37225	1.53009
H	-1.94817	-0.85044	2.52794
O	-2.37695	-1.44458	-3.31948
C	-3.42882	-2.02737	-3.76811
O	-3.23734	-2.93177	-4.76602
O	-4.60119	-1.77280	-3.31681
C	-4.50848	-3.70043	-5.28099
C	-4.87670	-4.95759	-4.60591
H	-5.29132	-2.92788	-5.23693
H	-5.10799	-4.80350	-3.53182
H	-4.20506	-3.85514	-6.33135
H	-4.09206	-5.73619	-4.69952
Li	-3.64114	-0.35322	-2.16238
O	-4.95941	-0.54984	-0.49206
O	-6.48042	0.16958	1.03219
C	-6.45658	1.12919	2.13493
C	-4.97931	1.15509	2.52316
O	-4.31902	0.71245	1.29638
C	-5.22670	0.06759	0.52675
H	-7.11636	0.75794	2.93010
H	-6.81901	2.09610	1.75103
H	-4.60378	2.15592	2.77392
H	-4.72985	0.43591	3.31946
O	-4.52525	1.34541	-3.01323
C	-5.48105	2.02483	-2.67540
O	-5.78225	2.34523	-1.39233
C	-7.10312	2.97235	-1.37485
C	-7.25602	3.46036	-2.81360
O	-6.36845	2.55955	-3.54973
H	-7.84080	2.20089	-1.10173
H	-7.09186	3.78070	-0.63218
H	-8.27173	3.34684	-3.21449
H	-6.89246	4.48963	-2.96185

C	-7.84468	-3.39332	-7.36322
O	-7.68736	-4.53237	-6.94534
O	-9.05297	-2.79369	-7.46053
O	-6.84443	-2.60113	-7.80200
C	-8.88464	-1.51261	-8.14509
C	-7.37931	-1.26128	-8.03397
H	-9.49680	-0.76283	-7.62644
H	-6.92837	-0.86602	-8.95370
H	-9.22621	-1.63778	-9.18486
H	-7.10468	-0.63483	-7.17100
O	-8.01621	-4.19745	-3.85255
C	-7.86216	-3.00184	-3.62953
O	-7.80709	-2.47443	-2.39205
O	-7.76263	-2.06261	-4.59487
C	-7.69641	-1.01971	-2.49223
C	-7.40085	-0.78506	-3.98032
H	-8.65594	-0.59145	-2.16167
H	-6.32827	-0.62878	-4.16550
H	-6.87195	-0.71023	-1.83598
H	-8.02555	-0.00601	-4.43788
Li	-6.93254	-5.29600	-5.21586
O	-7.66347	-7.14164	-5.20618
O	-9.84029	-7.11343	-5.87701
C	-10.83788	-8.14295	-6.16590
C	-9.98102	-9.37220	-6.47502
O	-8.71104	-9.05183	-5.82707
C	-8.66101	-7.71910	-5.61547
H	-11.44736	-7.80346	-7.01400
H	-11.46247	-8.26655	-5.26721
H	-10.36740	-10.30015	-6.03316
H	-9.78516	-9.50615	-7.54985
O	-6.07865	-7.47828	-8.67093
C	-7.11215	-6.94833	-9.03102
O	-8.35715	-7.36670	-8.66691
C	-9.33443	-6.39079	-9.13474
C	-8.56810	-5.64790	-10.23095
O	-7.17458	-5.88190	-9.87270
H	-10.21346	-6.93347	-9.50771
H	-9.60188	-5.74850	-8.28237
H	-8.73749	-6.07011	-11.23469
H	-8.74720	-4.56388	-10.24025

Supplementary References

1. Ding, K. *et al.* Preparation of CuBr nanoparticles on the surface of the commercial copper foil via a soaking method at room temperature: Its unexpected facilitation to the discharge capacity of the commercial graphite electrode. *J. Electroanal. Chem.* **877**, 114626 (2020).
2. Yang, H. *et al.* Designing Cation-Solvent Fully Coordinated Electrolyte for High-Energy Density Lithium-Sulfur Full Cell Based On Solid-Solid Conversion. *Angew. Chem. Int. Ed.* **60**, 17726-17734 (2021).
