# Supplementary material for: Suppressing electrolyte-lithium metal reactivity via Li+-desolvation in uniform nano-porous separator
Source: Nat Commun. 2022 Jan 10;13:172. doi: 10.1038/s41467-021-27841-0 (PMC8748786; doi:10.1038/s41467-021-27841-0)
Supplement: Supplementary file 3 — Description of Additional Supplementary Files [file 41467_2021_27841_MOESM3_ESM.docx]

Supplementary Information file (PDF)

Supplementary Video 1 │ In-situ optical microscopy measurement of the PP separator-based pouch cell to observe lithium deposition.

Supplementary Video 2 │ In-situ optical microscopy measurement of the Zr-MOCN-based pouch cell to observe lithium deposition.
