# Supplementary figures and images for: Suppressing electrolyte-lithium metal reactivity via Li+-desolvation in uniform nano-porous separator
Source: Nat Commun. 2022 Jan 10;13:172. doi: 10.1038/s41467-021-27841-0 (PMC8748786; doi:10.1038/s41467-021-27841-0)

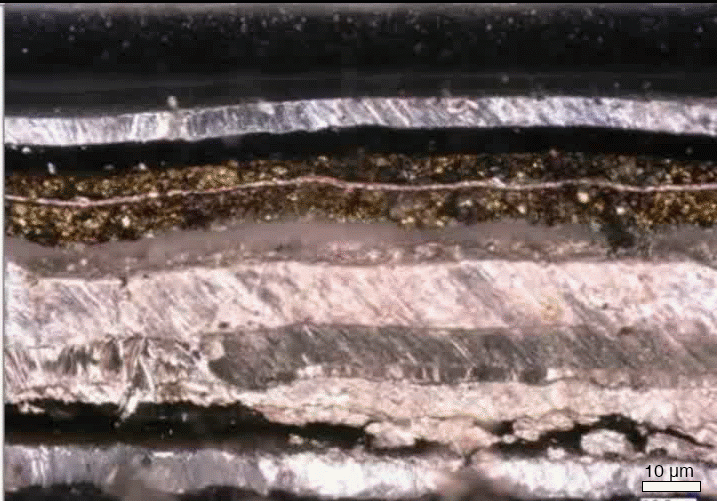

Supplement: Supplementary file 4 — Supplementary Video 1 [file 41467_2021_27841_MOESM4_ESM.gif]

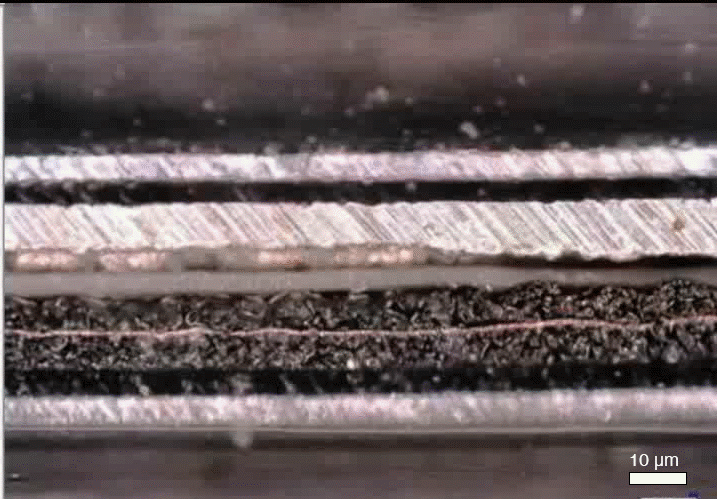

Supplement: Supplementary file 5 — Supplementary Video 2 [file 41467_2021_27841_MOESM5_ESM.gif]
